# Assessment of Gold Nanoparticles-Inhibited Cytochrome P450 3A4 Activity and Molecular Mechanisms Underlying Its Cellular Toxicity in Human Hepatocellular Carcinoma Cell Line C3A

**DOI:** 10.1186/s11671-018-2684-1

**Published:** 2018-09-10

**Authors:** Kyoungju Choi, Hyun Joo

**Affiliations:** Department of Anatomy & Physiology, Nanotechnology Innovation Center of Kansas State (NICKS), College of Veterinary Medicine, Kansas State University, Manhattan, KS 66506 USA

**Keywords:** Gold nanoparticles, Human hepatocellular carcinoma cell C3A, Oxidative stress, Hepatic transporters, Mitochondrial dysfunction

## Abstract

Interactions of the 40 and 80 nm gold nanoparticles (AuNP) functionalized with cationic branched polyethylenimine (BPEI), anionic lipoic acid (LA), or neutral polyethylene glycol (PEG) with human hepatocellular carcinoma (HCC) cell line C3A have been investigated in the absence and presence of human plasma protein corona (PC). All bare (no PC) AuNP besides 80 nm LA-AuNP were cytotoxic to C3A but PC attenuated their cytotoxicities. Time-dependent cellular uptake of AuNP increased besides 40 nm BPEI-AuNP but PC suppressed their uptakes besides 80 nm PEG-AuNP. Biphasic responses of oxidative/nitrosative stress by BPEI-AuNP occurred in C3A cells, whereas PEG-AuNP was a potent antioxidant. All bare AuNP inhibited cytochrome P450 (CYP) 3A4 activity irrespective of size and surface charge but PC recuperated its activity besides PEG-AuNP. The 40 nm PEG-AuNP-modulated gene expression was mainly involved in mitochondrial fatty acid β-oxidation and to a less degree hepatic efflux/uptake transporters. These studies contribute to a better understanding of AuNP interaction with key biological processes and their underlying molecular mechanisms in HCC, which may be further implicated in the development of more effective therapeutic target in HCC treatment.

**Electronic supplementary material:**

The online version of this article (10.1186/s11671-018-2684-1) contains supplementary material, which is available to authorized users.

## Background

Hepatocellular carcinoma (HCC) is one of the most common cancer worldwide and the fastest-growing cause of cancer mortality in the USA [1, 2]. Given that HCC has been diagnosed at advance stages, the curative HCC treatments include liver transplantation or surgical resection at the early tumor development and chemo- and radio-therapy for an advanced state of tumor. HCC often develops a high resistance to conventional antineoplastic agents, a non-selective cytotoxic molecule that could result in the systemic adverse effects. The recent advances in gene therapy, i.e., RNA interference (RNAi)-based gene therapy, have been utilized in the current HCC treatment [3, 4]. The efficacy of RNAi requires the vector to be delivered to the interior of the target cell [5]. The vectors for a successful gene delivery are viral and non-viral vectors. Viruses offer greater efficiency of gene delivery, but non-viral vectors are preferred due to safety concerns with the viral vectors. Nanoparticles (NP) as non-viral vectors for targeted gene delivery or drug delivery system have gained great attention for improving therapeutic efficiency and lowering toxicity on the systemic and/or cellular levels in HCC treatment [4, 6]. Thus, it becomes very important to identify the molecular mechanism and biological pathway underlying cellular disturbance and toxicity of NP in target cells and tissues. Recent in vitro studies demonstrated that gene expression profiling combined with cellular and biochemical responses has provided a direct assessment of cellular perturbation and potential NP toxicity [7–10].

The gold nanoparticles (AuNP) have been used as delivery vehicle for target-specific delivery of gene-silencing moieties, alone or in combination with other drugs due to their unique physicochemical properties and surface chemistries [11, 12]. AuNP interaction with blood plasma proteins forms protein corona, which in turn alters NP surface chemistries and influences the subsequent biological responses such as the cellular uptake and potential toxicity [13, 14]. Cellular uptakes of AuNP in different human cancer cell lines and primary cells were critically affected by protein corona formation, irrespective of size and surface charge [7–9, 14–17].

The size- and surface charge-dependent oxidative stress was also observed in human breast cancer cell line, MDA-MB-231, hepatocellular carcinoma HepG2, and human leukemia HL-60 cells in response to AuNP, which were associated with NP cytotoxicity [18, 19]. AuNP-induced cytotoxicity occurred in various human cancer cell lines and primary human cells in a cell type-specific manner [7–9, 20, 21].

Cytochrome P450 (CYP) enzymes play the important role in bioactivation or inactivation of numerous cytotoxic drugs, and host susceptibility to the carcinogenicity of anticancer drugs [22]. AuNP influenced the catalytic activity of CYP enzymes at the cellular and molecular levels in vivo and in vitro [7, 23–25]. AuNP has considerably exhibited the differential gene expression predominantly involved in oxidative stress markers in human lung fibroblast cell line MRC-5, and mitochondrial dysfunction in human umbilical vein cells (HUVEC) and human hepatocytes, which correlates with an increase in lipid peroxide production and a high cytotoxicity [8, 9, 26]. While this knowledge reciprocally suggests that AuNP causes apoptotic or necrotic cell death in various cell types and alters cellular and biochemical functions combined with differential gene expression in stress response pathways and toxicity, the specific pathways through which AuNP exerts their toxic effects within the cell or biological system remain unknown.

Herein, this study investigated the effects of protein corona, size, and surface charge on AuNP interaction with human HCC cell C3A. Primarily, time-dependent cellular uptake of the 40 and 80 nm AuNP functionalized with cationic BPEI, anionic lipoic acid (LA), or neutral polyethylene glycol (PEG) in C3A cells was determined with and without human plasma protein corona (PC). Secondly, AuNP-induced cytotoxicity and reactive oxygen species (ROS)/reactive nitrogen species (RNS) production were monitored along with their inhibitory effects on CYP3A4 activity. Lastly, AuNP toxicity-associated molecular mechanism of action was characterized using the Human Molecular Toxicology Pathway Finder and the Human Drug Transporters RT^2^ Profiler™ PCR array.

## Methods

### Gold Nanoparticles Synthesis

The 40 and 80 nm cationic BPEI, anionic LA, and neutral PEG Biopure™ AuNP was custom synthesized from nanoComposix (San Diego, CA). Particle size, polydispersity index (PDI), and zeta (z)-potential and spectral properties were characterized with dynamic light scattering (DLS), transmission electron microscopy (TEM), and UV-Vis spectroscopy. AuNP were synthesized through the reduction of hydrogen tetrachloroaurate (III) hydrate in potassium carbonate aqueous solution followed by the aging process and tangential flow filtration (TFF). AuNP surface was functionalized with LA or PEG by adding dihydrolipoic acid (0.2:1, *w*/*w*) or thiol-methoxy-terminated PEG (Laysan Bio Inc., Arab, AL) (0.5:1, *w*/*w*), respectively, followed by TFF washing and sterile filtration. BPEI-functionalized surfaces of AuNP were synthesized via EDC chemistry by linking the carboxylic acid of LA to free amines of BPEI followed by TFF washing and subsequent centrifugation for a removal of unbound BPEI.

### Protein Corona Preparation

Pooled human blood plasma (*n* = 5) were obtained from the Biological Specialty Corp. (Colmar, PA). AuNP were incubated in human plasma (55%, *v*/*v*) at a constant speed of 250 rpm at 37 °C for 1 h as reported [7, 8]. The unbound and weakly associated proteins were removed by repeated washes with phosphate-buffered saline (PBS) at 20,000×*g* for 20 min at 20 °C. The final human plasma protein corona (PC)-coated AuNP were dispersed in PBS and then diluted in cell culture medium for further physicochemical characterization or dosing. The detailed protocol is given in the Additional file 1.

### Physicochemical Characterization of AuNP

Hydrodynamic diameters (*D*_H_), PDI, and z-potential of the 40 and 80 nm bare (no PC) AuNP functionalized with BPEI, LA, and PEG in deionized (DI) water were analyzed at 25 °C at 0 h using the Zetasizer Nano ZS (Malvern Instruments, Worcestershire, UK); for PC-coated AuNP in PBS at 25 °C at 0 h; and for all bare and PC AuNP in complete cell culture medium at 37 °C at 0 h and 24 h. Complete cell culture medium contained Eagle’s minimum essential medium (EMEM) supplemented with 10% FBS (ATCC^®^, Manassas, VA). A sample was measured 5 times with 11 sub-runs of 10 s each. In addition, optical absorption spectra were measured using the Synergy H1 hybrid multi-mode microplate reader (BioTek Instruments Inc., Winooski, VT) at room temperature at 0 h.

### Transmission Electron Microscope

The AuNP morphology was characterized using TEM. All bare and PC AuNP solution (5 μL) was placed on 200 mesh copper grids followed by air-drying at room temperature. The samples were viewed on a Tecnai G2 Spirit BioTWIN with an Oxford detector (FEI Company, Hillsboro, OR) at an accelerating voltage of 120 kV. The GATAN microscopy suite (GATAN Inc., Pleasanton, CA) measured AuNP diameters.

### Cell Culture and Viability Measurement

Human hepatocellular carcinoma C3A cells (ATCC^®^CRL-10741™) were purchased from ATCC^®^ (Manassas, VA), cultured in complete EMEM (ATCC^®^, Manassas, VA) supplemented with 10% FBS, and expanded to approximately 80% confluence in T75 flask with medium changes every 4 days. After 0.25% (*w*/*v*) trypsin–0.53 mM ethylenediaminetetraacetic acid (EDTA) digestion, cells were plated in 96-well plates at 8 × 10^4^ cells per well and incubated at 37 °C in a humidified atmosphere of 95% air and 5% CO_2._ After 48-h incubation, cells were dosed with AuNP in the absence and presence of PC. The C3A cells between passage 9 and 12 were used for the dosing.

The C3A viability was determined using the alamarBlue^®^ viability assay (Thermo Sci., Waltham, MA) as described [7, 27]. Cells in the 96-well plates were treated with the 40 and 80 nm BPEI-, LA-, and PEG-AuNP with and without PC ranging from 0 to 250 μg/cm^2^. After 24 h, 10% of alamarBlue^®^ reagent in complete EMEM (*v*/*v*) was added to the cell culture and incubated for 3 h at 37 °C. The complete EMEM served as a dispersant. The interactions of AuNP with the active ingredient of alamarBlue^®^ reagent, resazurin or a reduced product, resorufin were measured as controls. AuNP and resazurin (no cells) or maintenance medium (no cells) served as background controls. Fluorescence, proportional to cell viability, was normalized to controls and expressed as a percentage relative to control cell group.

### Cellular Uptake Measurement with Inductively Coupled Plasma Mass Spectrometry

Cells were seeded at 8 × 10^4^ cells per well of 96-well plates and dosed with nontoxic concentration of 1.56 μg/cm^2^ of all bare and PC AuNP for 0.5, 1, 3, 6, 12, and 24 h. The etching step was incorporated to remove cell membrane-bound AuNP and its non-specific binding to the wells as previously reported [28]. Cell harvest was dried and digested in aqua regia and intracellular Au concentration was quantified using the NexION^™^ 350X inductively coupled plasma mass spectrometry (ICP-MS) (PerkinElmer, Waltham, MA). Cellular uptake of AuNP was calculated as previously reported and expressed as the number of AuNP per cell [29]. The detailed protocol is given in the Additional file 1.

### Oxidative/nitrosative stress measurements

Cells were seeded at 8 × 10^4^ cells per well of 96-well plates and dosed with the 40 nm bare BPEI- and PEG-AuNP up to 125 μg/cm^2^ for 1, 3, and 24 h. Direct measurement of oxygen/nitrosative stress was assayed with total reactive oxygen species (ROS)/superoxide (SO) assay kit (Enzo Life Sciences, Farmingdale, NY) as previously described [30]. Fluorescence, proportional to the increase in ROS/reactive nitrogen species (RNS) (Ex488/Em520 nm) or SO (Ex550/Em610 nm) were measured with the microplate reader. The detailed protocol is given in the Additional file 1.

### Cytochrome P450 3A4 Activity

Adverse effects of the 40 and 80 nm bare and PC AuNP on CYP3A4 activity was characterized using P450-Glo™ assays (Promega Corp., Madison, WI) as fully described [7]. C3A cells in 96-well plates were dosed at the median lethal concentration (LC_50_) values: 127.3 μg/cm^2^ of the 40 nm BPEI-AuNP, 205.5 μg/cm^2^ of the 80 nm BPEI-AuNP, 192.5 μg/cm^2^ of the 40 nm LA-AuNP, and 129.5 μg/cm^2^ of the 40 nm PEG-AuNP. Since LC_50_ values of the 80 nm LA- and PEG-AuNP were not determined, cells were treated with LC_50_ values of the 40 nm LA- and PEG-AuNP (192.5 μg/cm^2^ and 129.5 μg/cm^2^, respectively). After the end of 24-h incubation, cells were incubated with a CYP3A4 substrate (luciferin-IPA) at 37 °C for 3 h. Luminescence signal, proportional to an enzyme activity, was measured with a microplate reader and then normalized to controls. Controls were assigned to assess the interaction of AuNP with parent substrates or metabolites and cell-free substrates. CYP activity was expressed as a percentage relative to control cell group.

### Gene Expression Profiling

Since toxic 40 nm PEG-AuNP were employed in the inhibition of CYP3A4 activity and antioxidant activity in C3A cells showing high cellular uptake, it was selected to characterize molecular mechanisms of action underlying its toxicity and differential cellular responses. Cells were seeded at 2.5 × 10^6^ cells per well of 6-well plates and dosed with LC_50_ value of the 40 nm PEG-AuNP for 24 h at 37 °C. At the end of incubation, cells were subject to RNA isolation and then cDNA synthesis using total RNA with an average RNA integrity numbers (RIN) value of 7.8 was conducted as previously described [7–9]. The resulting cDNA was mixed with RT^2^ SYBR green master mix (Qiagen Inc., Valencia, CA) and then applied to the Human Molecular Toxicology Pathway Finder or Human Drug Transporters RT^2^ Profiler™ PCR arrays in Quantstudio™ 7 Flex (Applied BioSystem, Foster City, CA). Differentially expressed genes with the fold change <− 2 and > 2 and a *p* < 0.05 represented down- and up-regulation of the gene of interest. To validate the RT^2^ PCR array data, an expression of nine selected genes was evaluated with cDNA synthesis and subsequent real-time PCR. Primer sequences are summarized in Additional file 1: Table S1. All PCR reactions were performed in triplicate. The detailed protocol of real-time PCR conditions and quantification is given in the Additional file 1.

### Statistical Analysis

Median lethal concentration (LC_50_) values of AuNP in C3A cells were estimated by fitting a Hill equation with variable slope to the observed data (the input of AuNP concentration levels and the corresponding cell viability) using GraphPad Prism 6 (La Jolla, CA) as described [7]. One-way analysis of variance (ANOVA) was conducted using SAS 9.4 (SAS Institute, Cary, NC) to assess the effects of AuNP on ROS/RNS production and cellular uptake in C3A cells. If significant, the multiple comparison was performed with Tukey’s honest significant difference (HSD) test at a *p* < 0.05.

## Results and Discussion

### Physicochemical Characterization of Bare and Human Plasma PC AuNP

**Fig. 1:**
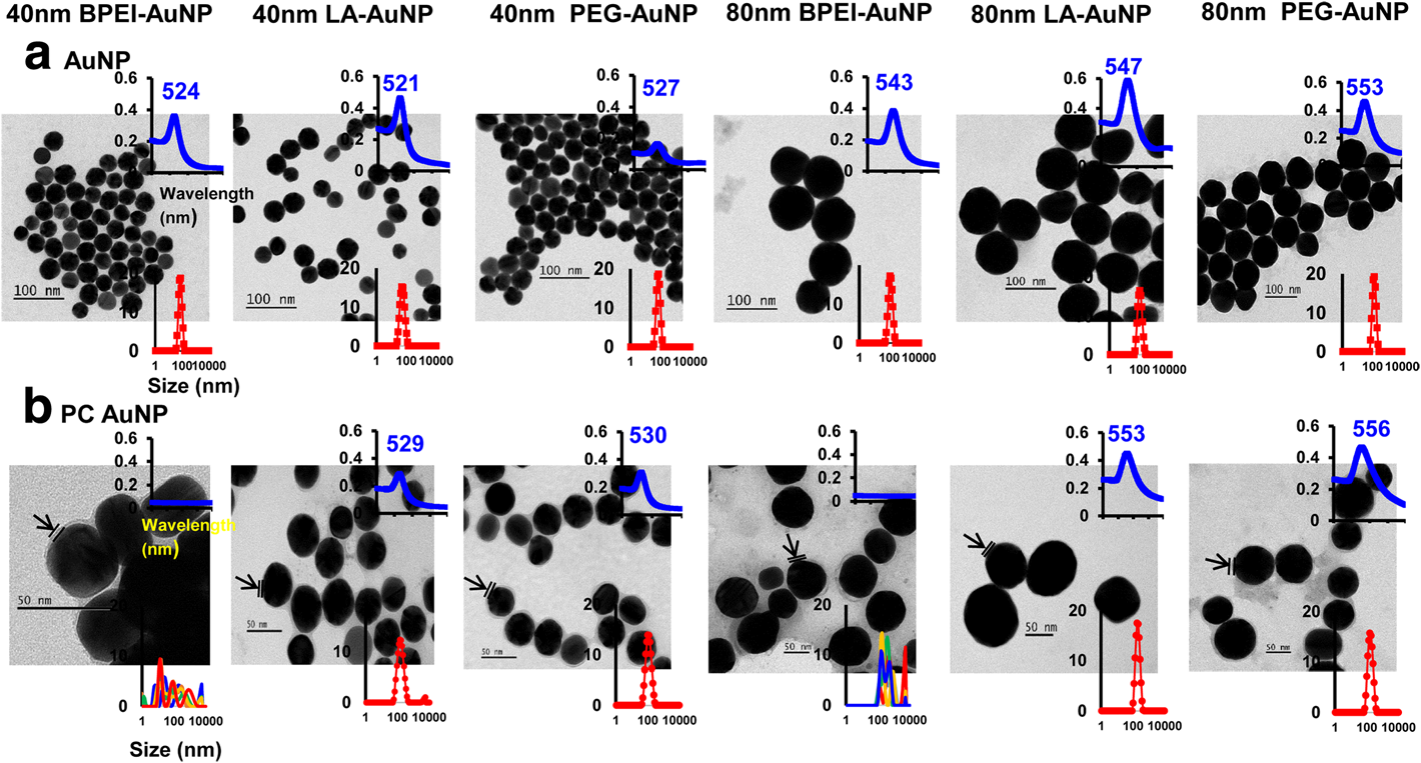
Transmission electron micrographs of **a** AuNP in deionized water and **b** PC AuNP in PBS at 0 h at 25 °C, UV-Vis spectra wavelength (upper inset), and the dynamic light scattering distribution (lower inset). Arrows indicate PC formation. *PC* human plasma protein corona, *BPEI* branched polyethylenimine, *LA* lipoic acid, *PEG* polyethylene glycol

The effects of NP size, surface charge, and human plasma PC formation around AuNP on hydrodynamic diameter (D_H_), polydispersity index (PDI), z-potential, and a spectral property as well as the morphology have been characterized using DLS, TEM, and UV-Vis spectroscopy (Fig. 1). In TEM images, all bare (no PC) AuNP in DI water was monodisperse with the tight size distribution and unique UV-Vis spectrum ranges of 521–553 nm (Fig. 1a). PC formations around AuNP were observed with the changes in size distribution and redshifts of the absorption spectra (Fig. 1b). The *D*_H_ and PDI values of the 40 and 80 nm bare and PC AuNP in complete EMEM were compatible up to 24 h at 37 °C except for the 40 and 80 nm PC BPEI-AuNP which showed a decrease in PDI values (0.29 and 0.32, respectively) at 24 h at 37 °C compared to those at 0 h at 37 °C (0.62 and 1.0, respectively) (Table 1). Z-potential values of all bare and PC AuNP relatively decreased at 24 h at 37 °C compared to those at 0 h at 37 °C. An aggregation of the 40 and 80 nm PC BPEI-AuNP in PBS and complete EMEM was observed, which correlated with multiple peaks in a size distribution and changes in D_H_ and redshifts of absorbance spectra relative to bare BPEI-AuNP (Fig. 1 and Additional file 1: Figure S1, Table 1). These results were supported by the recent studies that the 40 and 80 nm PC and human serum albumin corona-coated BPEI-AuNP were aggregated in PBS and various cell culture medium [7–9].

**Table 1:** The D_H_, PDI, and z-potential of 40 and 80 nm bare and PC AuNP with BPEI, LA, and PEG in medium* at 37 °C. Data is mean ± SD (*n* = 5)

AuNP size (nm)	Surface coating	D_H_ (nm)		PDI		z-potential	
		0 h	24 h	0 h	24 h	0 h	24 h
40	BPEI	227.9 ± 6.0	193.5 ± 2.7	0.15 ± 0.01	0.22 ± 0.01	− 9.3 ± 1.0	− 10.8 ± 1.4
	LA	65.9 ± 0.2	86.7 ± 0.5	0.26 ± 0.01	0.22 ± 0.01	− 10.2 ± 2.0	− 11.3 ± 1.3
	PEG	72.0 ± 0.5	76.5 ± 0.2	0.25 ± 0.01	0.24 ± 0.01	− 9.4 ± 1.5	− 10.1 ± 0.7
	PC BPEI	ND	ND	0.62 ± 0.01	0.29 ± 0.01	− 10.5 ± 0.5	− 10.1 ± 0.3
	PC LA	81.3 ± 0.3	87.3 ± 0.38	0.14 ± 0.01	0.10 ± 0.01	− 8.4 ± 0.8	− 10.8 ± 0.6
	PC PEG	129.0 ± 2.6	117.6 ± 3.22	0.28 ± 0.01	0.18 ± 0.01	− 7.9 ± 0.6	− 9.9 ± 0.9
80	BPEI	205.9 ± 3.4	196.1 ± 6.7	0.13 ± 0.02	0.13 ± 0.02	− 9.0 ± 1.3	− 10.1 ± 0.6
	LA	136.1 ± 0.5	144.6 ± 1.3	0.10 ± 0.01	0.10 ± 0.01	− 10.5 ± 1.2	− 10.5 ± 2.2
	PEG	128.9 ± 0.5	127.9 ± 0.6	0.04 ± 0.01	0.05 ± 0.02	− 2.3 ± 0.3	− 7.6 ± 0.6
	PC BPEI	ND	ND	1.00 ± 0.06	0.32 ± 0.01	− 9.4 ± 0.4	− 10.8 ± 0.7
	PC LA	127.7 ± 0.9	139.7 ± 1.2	0.10 ± 0.01	0.08 ± 0.02	− 9.1 ± 0.9	− 10.9 ± 0.5
	PC PEG	139.6 ± 0.3	134.5 ± 1.8	0.05 ± 0.01	0.05 ± 0.02	− 3.6 ± 0.6	− 4.7 ± 0.5

*D*_*H*_ hydrodynamic diameter, *PDI* polydispersity index, *BPEI* branched polyethylenimine, *LA* lipoic acid, *ND* not determined, *PEG* polyethylene glycol, *PC* human plasma protein corona; Bare, no PC; *, Eagle’s minimum essential medium (EMEM) supplemented with 10% FBS (ATCC^®^, Manassas, VA)

### AuNP Cytotoxicity

**Fig. 2:**
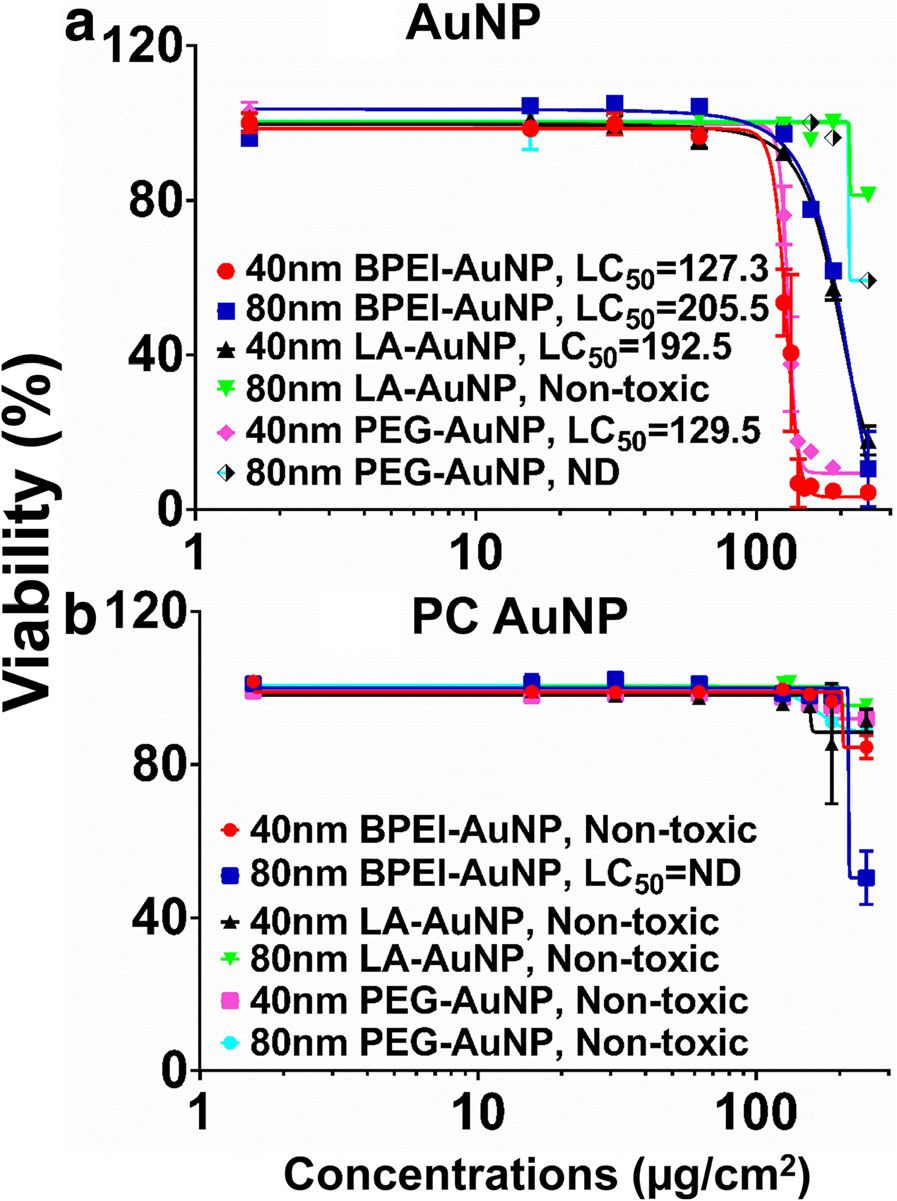
C3A viability and LC_50_ values of the 40 and 80 nm **a** AuNP and **b** PC AuNP. Data represent mean ± S.D. (*n* = 3). PC human plasma protein corona, ND not determined, BPEI branched polyethylenimine, LA lipoic acid, PEG polyethylene glycol, LC_50_ median lethal concentration

AuNP cytotoxicity was measured using median lethal concentration (LC_50_) in C3A cells. The NP surface charge-, particle size-, and PC formation around NP-dependent LC_50_ analyses with AuNP were shown in Fig. 2. All 40 nm BPEI-, LA, and PEG-AuNP and the 80 nm BPEI-AuNP were cytotoxic to C3A cells with the corresponding LC_50_ ranges from 127.3 to 205.5 μg/cm^2^ (Fig. 2a). The 80 nm bare PEG-AuNP exhibited 59% cell viability at the highest concentration of 250 μg/cm^2^, whereas the 80 nm LA-AuNP were not cytotoxic. PC reduced AuNP toxicity as function of size and surface charge modification except for the 80 nm BPEI-AuNP which showed 51% cell viability at 250 μg/cm^2^ at 24 h (Fig. 2b). Recent studies demonstrated that the 40 nm bare BPEI-AuNP were toxic to primary human hepatocytes, HUVEC, and human renal proximal tubular cells (HPTC) (LC_50_ ranges as 22.4–80.3 μg/cm^2^) [7–9]. The PC-coated BPEI-AuNP were cytotoxic to human hepatocytes, but HSA-coated AuNP were not cytotoxic [7]. These results suggested that C3A cells are more resistant to AuNP toxicity than primary human cells due to a high proliferation rate and metabolic activity of cancer cell line [31].

### Intracellular Uptake of AuNP

**Fig. 3:**
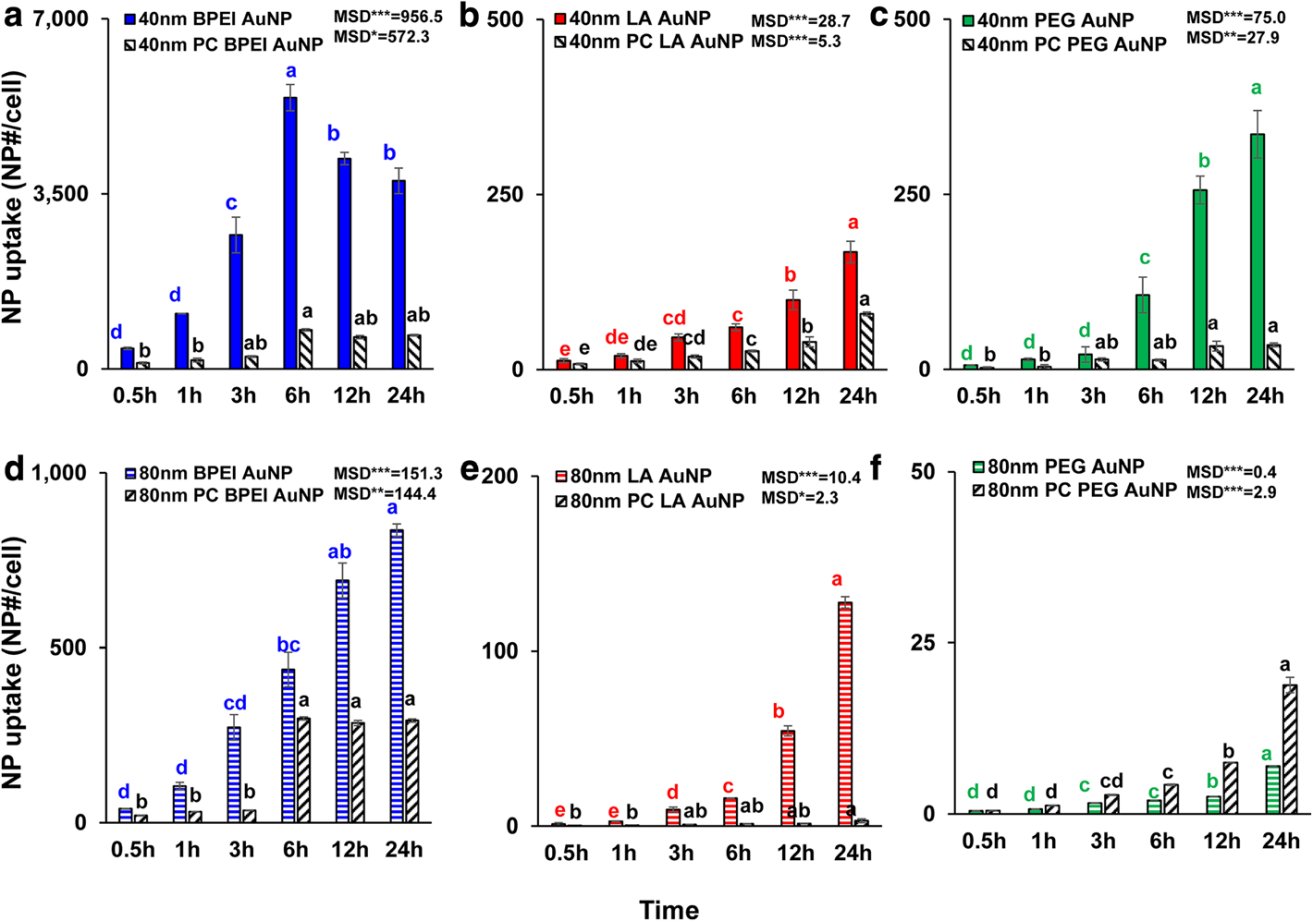
Time-dependent cellular uptake of the 40 nm **a** BPEI-AuNP, **b** LA-AuNP, and **c** PEG-AuNP, and the 80 nm **d** BPEI-AuNP, **e** LA-AuNP, and **f** PEG-AuNP in the absence and presence of PC in C3A cells up to 24 h. Data represent mean ± S.D. (*n* = 3). Letters were significantly different according to Tukey’s HSD test. *BPEI* branched polyethylenimine, *LA* lipoic acid, *PEG* polyethylene glycol, *PC* human plasma protein corona, *MSD* minimum significant difference. *p < 0.05; ** *p* < 0.005; ***p < 0.0001

NP size-, surface charge-, and PC-dependent cellular uptake of all bare and PC AuNP were determined at 1.56 μg/cm^2^ up to 24 h. ANOVA showed significant changes with size, PC and time (*p* < 0.0001), and interactions (PC × size, PC × time, size × time, and PC × size × time) (*p* < 0.001) for all AuNP uptake besides an insignificant interaction (PC × size) for LA- and PEG-AuNP uptake (*p* = 0.2). As shown in Fig. 3a–f, a linear increase in cellular uptake of the 40 and 80 nm bare and PC AuNP was observed besides the 40 nm bare and PC BPEI-AuNP which reached the highest cellular uptake at 6 h and decreased afterward (Fig. 3a). However, at 24 h, the 40 nm cationic BPEI-AuNP contained the highest uptake followed by neutral 40 nm PEG-AuNP and then anionic 40 nm LA-AuNP, which was associated with the order of C3A cell cytotoxicity of AuNP (Fig. 2a). This result is consistent with the previous studies that cationic poly (N-(2-aminoethyl) acrylamide)- and BPEI-AuNP had the greatest cellular uptake compared to those of anionic poly(acrylic acid)- and LA-AuNP and neutral poly(N-(2,3-dihydroxypropyl)acrylamide- and PEG-AuNP in human colorectal adenocarcinoma Caco-2 cells, HPTC, and human hepatocytes [9, 32]. In addition, NP-PC complex attenuated all 40 and 80 nm BPEI- and LA-AuNP and the 40 nm PEG-AuNP in C3A cells but accelerated the 80 nm PEG-AuNP uptake (Fig. 3f). These results are supported by the recent studies that PC inhibited AuNP uptake in HUVEC, HEK, and HPTC, irrespective of size and surface charge [8, 9, 33]. In contrast, PC and HSA coronas enhanced the 40 nm PEG-AuNP uptake in human hepatocytes but that latter induced the 80 nm PEG-AuNP uptake in HEK [7, 33].

### Oxidative and Nitrosative Stress Measurements

**Fig. 4:**
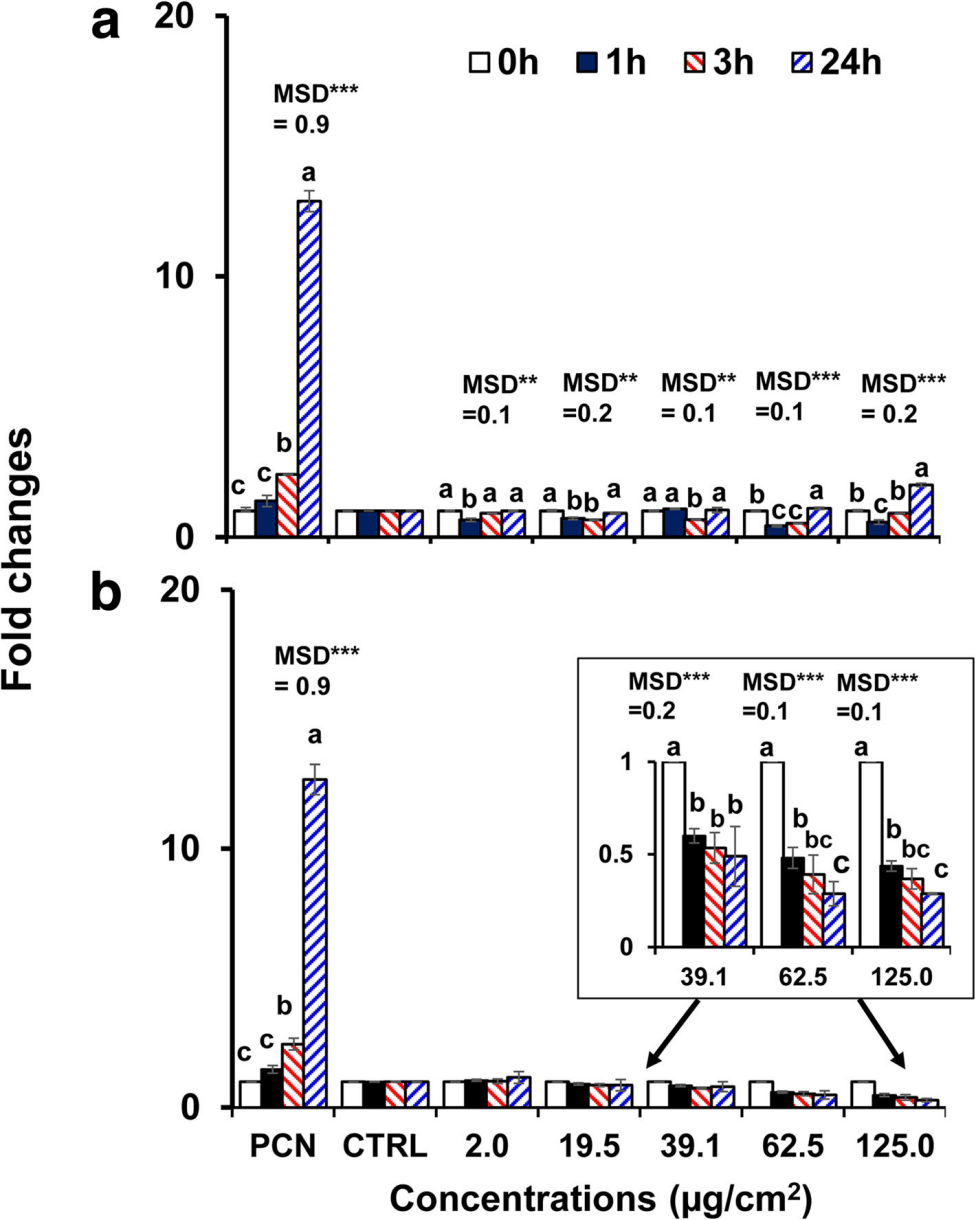
Time- and concentration-dependent ROS/RNS production in C3A cells exposed to **a** the 40 nm BPEI-AuNP and **b** the 40 nm PEG-AuNP up to 24 h. Data represent mean ± S.D. (*n* = 3). Letters were significantly different according to Tukey’s HSD test. *BPEI* branched polyethylenimine, *LA* lipoic acid, *PEG* polyethylene glycol, *CTRL* control, *MSD* a minimum significant difference, *PCN* pyocyanin (ROS inducer). ** *p* < 0.005; ***p < 0.0001

Since the 40 nm bare BPEI- and PEG-AuNP exhibited the higher cytotoxicity and cellular uptake in C3A cells compared to other AuNP, they were selected to investigate AuNP-induced oxidative/nitrosative stress. Both AuNP modulated ROS/RNS generation in C3A cells in time- and concentration-dependent manner (*p* < 0.0001) and by interaction (time × concentration, *p* < 0.0001). As shown in Fig. 4a, ROS/RNS generation decreased at the higher concentrations of the 40 nm BPEI-AuNP (62.5 μg/cm^2^ and 125 μg/cm^2^) at 1 h at 37 °C but increased onward up to 24 h. In contrast, the 40 nm PEG-AuNP substantially suppressed ROS/RNS generation at 39.1 μg/cm^2^ onward with a fold change < 0.5 up to 24 h (Fig. 4b). Activation of cell death often contributes to NP toxicity and in most cases, an increase in ROS/RNS production, leading to oxidative stress, is responsible for NP toxicity [34]. The surface charge-dependent ROS/RNS production was observed with the 40 nm cationic BPEI- and neutral PEG-AuNP. The 40 nm BPEI-AuNP showed a biphasic pattern of ROS/RNS generation (antioxidant at 1 h and pro-oxidant at 3 h onward) at high concentrations, which was associated with its cytotoxicity in C3A cells (Fig. 2a). This result is consistent with the previous studies that the 40 and 80 nm BPEI-AuNP and the 20 nm citrate-AuNP-induced ROS generation was associated with their cytotoxicities in human hepatocytes and HepG2 cells, respectively, in time- and concentration-dependent manner [7, 35]. AuNP showed oxidative stress-induced cytotoxicity in human promyelocytic leukemia cells, HL-60 with a total glutathione reduction, irrespective of size [19]. In contrast, the 40 nm PEG-AuNP served as an antioxidant suggesting that oxidative/nitrosative stress may be not a direct mechanism of the 40 nm PEG-AuNP-induced cytotoxicity in C3A cells (Fig. 2b).

### CYP3A4 Activity Measurement

**Fig. 5:**
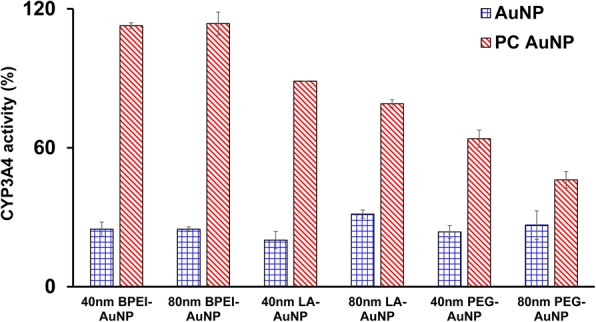
An inhibitory effect of AuNP on CYP3A4 activity in C3A cells exposed to the 40 and 80 nm BPEI-, LA-, and PEG-AuNP in the absence and presence of PC for 24 h. Values represent mean ± S.D. (*n* = 3). *BPEI* branched polyethylenimine, *LA* lipoic acid, *PEG* polyethylene glycol, *PC* human plasma protein corona

Inhibitory effects of the 40 and 80 nm bare and PC AuNP on CYP3A4 activity were characterized. As shown in Fig. 5, the 40 nm BPEI-, LA-, and PEG-AuNP and the 80 nm BPEI-AuNP at LC_50_ values inhibited the catalytic activity of CYP3A4 in C3A cells with the corresponding activity of 20.1 to 31.4% relative to the controls, irrespective of size and surface charge. Nontoxic concentrations of the 80 nm LA- and PEG-AuNP also suppressed its activity (31.4 and 26.6%, respectively). However, PC extensively ameliorated the 40 and 80 nm AuNP-induced CYP3A4 inhibition besides the 40 and 80 nm PEG-AuNP showing 63 and 46% activity compared to the controls. This is consistent with in vitro studies with human liver tissue and hepatocytes that anionic tannic acid-AuNP and cationic 40 and 80 nm BPEI-AuNP substantially inhibited the catalytic activity of CYP3A4 [7, 25]. In contrast, cationic PEI-AuNP and neutral polyvinylpyrrolidone-AuNP-induced mRNA expression of CYP1A2, CYP2C9, and CYP3A4 in HepG2 cells and CYP2B and CYP3A in rat liver slice, respectively [36, 37]. Recent study reports that the 40 and 80 nm bare and PC BPEI-AuNP substantially suppressed CYP3A4 activity in human hepatocytes via a conformational change in protein or blocking the substrate pocket as a reversible inhibition [7].

### Toxicity Pathway Focused Gene Expression Profiling of the 40 nm PEG-AuNP

**Fig. 6:**
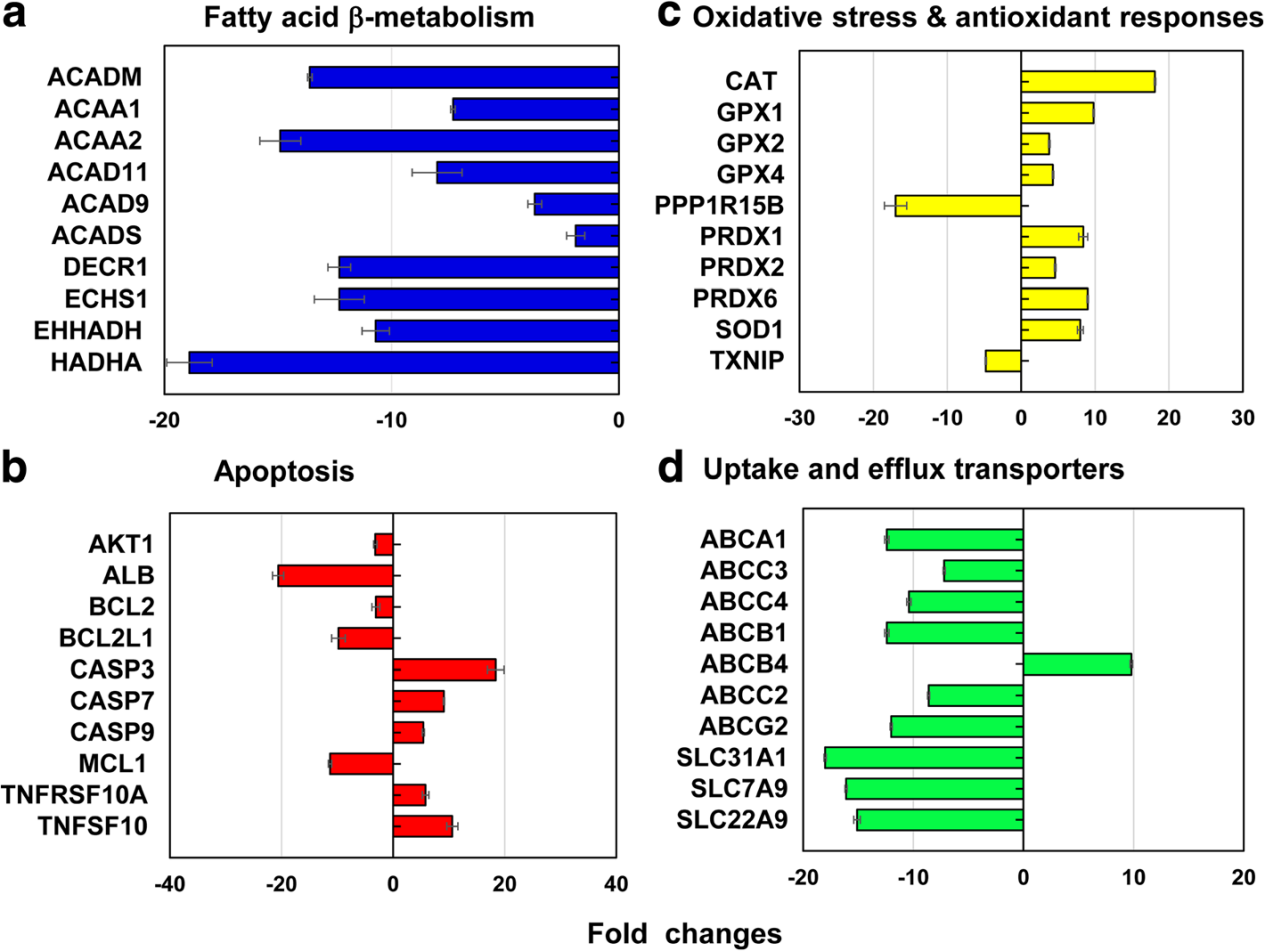
Representative genes involved in **a** mitochondrial fatty acid β-oxidation, **b** apoptosis, **c** oxidative stress and antioxidant responses, and **d** hepatic uptake and efflux transporters at LC_50_ value of 40 nm PEG-AuNP. All data had a fold change <− 2 and > 2 at *p* < 0.05. Gene ontology analysis is listed in Additional file 1: Tables S2–S7

From the representative genes covering 13 different stress and toxicity pathways, a total of 212 genes (↓186 and ↑26 genes) was differentially expressed at LC_50_ value of the 40 nm PEG-AuNP (Fig. 6, Additional file 1: Tables S2–S7). The 12.3% (26 genes, ↓26, ↑0 genes) of the total genes (212 genes) were predominantly involved in mitochondrial fatty acid β-oxidation; for apoptosis 11.3% (24 genes, ↓18, ↑6 genes); for DNA damage and repair pathway 11.3% (24 genes, ↓18, ↑6 genes); and for heat shock response 11.3% (24 genes, ↓22, ↑2).

In mitochondrial fatty acid β-oxidation pathway, genes encoding three different enzymes involved in the production of acyl-CoA and reducing equivalents of NADH and FADH_2_ were mainly suppressed; ACAD11, ACAD9, ACADM, and ACADS genes in Acyl-CoA dehydrogenases (2.0- to 13.6-fold); ACAA1 and ACAA2 in ketoacyl-CoA thiolases (7.3- to 14.9-fold); DECR1, ECHS1, EHHADH, and HADHA (10.7- to 18.9-fold) in enoyl-CoA hydratase (Fig. 6a, Additional file 1: Table S2). Mitochondrial fatty acid β-oxidation plays an important role in the production of acyl-CoA and reducing equivalents of NADH and FADH_2_, which is associated with four main enzymes (acyl-CoA dehydrogenases, enoyl-CoA hydratases, hydroxyacyl-CoA dehydrogenases, and ketoacyl-CoA thiolases [38, 39]. Further, electron carriers, NADH and FADH_2_, are involved in tricarboxylic acid (TCA) cycle and the mitochondrial respiratory chain, resulting in ATP production. In the current study, the 40 nm PEG induced mitochondrial dysfunction, a loss of ATP maintenance via a decrease in intracellular levels of ATP and FADH_2_, consequently defining its cytotoxicity in C3 cells (Fig. 2a). The similar phenomenon was reported in human hepatocytes, HUVEC and HPTC, exposed to the 40 nm BPEI-AuNP indicating that mitochondrial dysfunction may be a common mechanism of AuNP toxicity, irrespective of surface charge and cell types [7–9]. A recent study has reported that mitochondrial dysfunction-relevant cytotoxicity was observed in immortalized prostate cancer epithelial cells and lung cancer epithelial cells in response to an inhibitor of STAT3 phosphorylation, OPB-51602 [40].

In apoptosis pathway, the six pro-apoptotic genes of CASP3, CASP7, CASP9, TNFRSF10A, TNFRSF10B, and TNFSF10 were upregulated, whereas the six anti-apoptotic genes of AKT1, ALB, BCL2, BCL2L1, MCL1, and XIAP were downregulated (Fig. 6b, Additional file 1: Table S3), which was correlated with dose-dependent cytotoxicity in C3A cells (Fig. 2a). In DNA damage and repair check point, genes of the checkpoint kinases (CHEK1/2), the DNA excision repair genes (ERCC1/2/3), and the DNA ligase IV (LIG4) were upregulated but other excision repair genes (ERCC5/6, XRCC1/5), the checkpoint kinase (CDKN1A), and protein kinases (PRKDC) genes were downregulated (2- to 19-fold). These results suggested that the 40 nm PEG-AuNP-induced interference with cell cycle and DNA repair system may correlate with an induction of cell death in C3A cells (Fig. 2a, Additional file 1: Table S3). Genes encoding two different heat shock proteins (HSP) (A1A and A1B) were upregulated (10.2- to 14.2-fold) but HSP40 subfamily A, B, and C; HSP90 member 1; and HSP60 were downregulated (2- to 16-fold) (Additional file 1: Table S4).

In oxidative stress and antioxidant response, the 40 nm PEG-AuNP at LC_50_ value induced antioxidants genes and suppressed pro-oxidants, which was associated with a decrease in ROS/RNS generation being antioxidant itself (Fig. 4b). In antioxidant genes, glutathione peroxidase (GPX) 1, GPX2, GPX4, PRDX1, PRDX2, PRDX6, superoxide dismutase (SOD) 1, and CAT were induced (3.8- to 18.1-fold). In pro-oxidant genes, TXNIP and PPP1R15B were suppressed (4.8- and 17-fold, respectively) (Fig. 6c, Additional file 1: Table S5). This is consistent with a previous study that AuNP displayed oxidative stress-induced cytotoxicity in HepG2 and human hepatocytes, irrespective of size [7, 19].

In phase I metabolism, CYP3A4 and ESD genes were extensively suppressed (7-fold and 12-fold, respectively). Especially, inhibitory effect of 40 nm PEG-AuNP on CYP3A4 expression was correlated with a decrease in CYP3A4 activity (Fig. 5). Recent studies reported that the 40 nm BPEI-AuNP inhibited gene expression of CYP1A2, CYP2C9, and CYP3A4 in human hepatocytes; ESD in HUVEC; and CYP1A1 in HPTC [7–9]. Epidemiology study demonstrated that CYP enzymes in liver tissue of HCC patient were substantially inhibited by the tumorigenic process at the molecular and the functional level [41].

### Drug Uptake and Efflux Transporter Gene Expression Profiling

The development of multidrug resistance (MDR) by tumor cells is one of the main causes of cancer treatment failures [42, 43]. Integral membrane transporters-mediated decrease in drug uptake and increase in drug efflux including P-glycoprotein (P-gp) and breast cancer resistance protein (BCRP) is one of the major mechanisms of MDR.

**Fig. 7:**
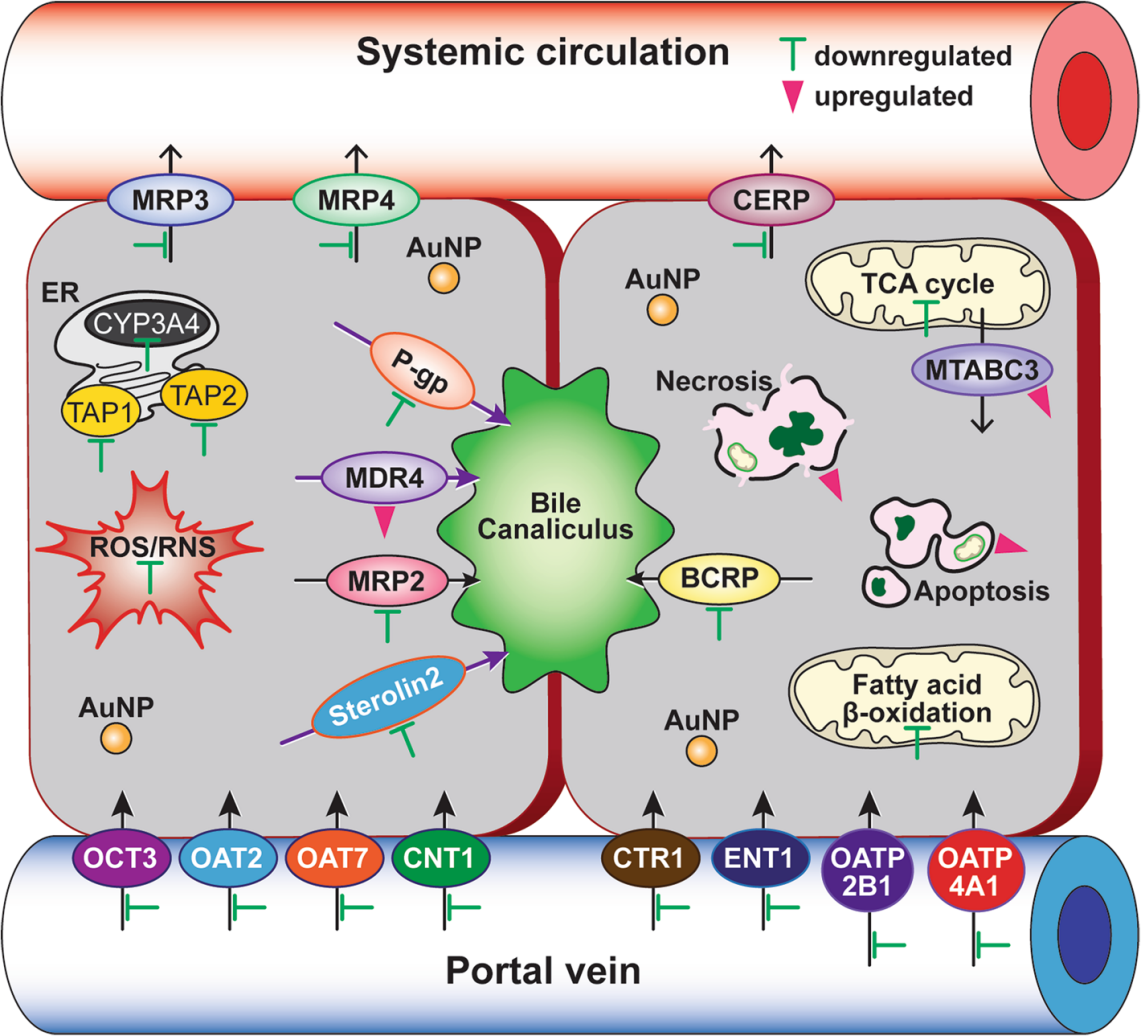
A schematic representation of the basic mechanisms of action of 40 nm PEG-AuNP in HCC treatment. Green bars (an inhibition) and pink triangles (an induction) indicate the 40 nm PEG-AuNP-modified biological markers and pathways. Gene ontology analysis is listed in Additional file 1: Tables S2–S7

Differential gene expression of drug efflux and uptake transporters in C3A cells exposed to 40 nm PEG-AuNP showed that a total of 14 genes of ABC transporters (↓12 and ↑2 genes) and a total of 21 genes of SLC transporters (↓21 and ↑0 genes) were substantially modulated at LC_50_ value (Figs. 6d and 7, Additional file 1: Table S7). In drug efflux transporters of ABC family, genes of multidrug resistance-associated protein (MRP3/ABCC3), MRP4 (ABCC4), and cholesterol efflux regulated protein (CERP/ABCA1) in basolateral membrane were downregulated (7.2- to 10.4-fold). The genes encoding P-gp (ABCB1), MRP2 (ABCC2), BCRP (ABCG2), and sterolin 2 (ABCG8) in canalicular efflux transporters were also suppressed (8.6- to 13.8-fold). In contrast, multidrug resistance (MDR4/ABCB4) in canalicular membrane and mitochondria ABC transporter (MTABC3/ABCB6) in the outer mitochondrial membrane were highly upregulated (9.8-fold and 5.8-fold, respectively). In drug uptake transporters, genes of copper transporter protein (CTR1/SLC31A1) and to a lesser degree organic anion transporting (OAT7/SLC22A9) were also inhibited (18- fold and 15-fold, respectively). These results support a recent study that the 40 nm BPEI-AuNP downregulate MDR3 in human hepatocytes but upregulates MRP3 in HUVEC indicating surface charge- and cell type-dependent interaction between AuNP and efflux transporters [7, 8]. Epidemiology study exhibited that a high expression of BCRP and a low expression of OCT3 occurred in HCC tumor, which was closely associated with the tumor progression and its size [44]. A previous study exhibited that P-gp inhibitor, verapamil enhanced cytotoxicity of glutathione-AuNP conjugated with doxorubicin in feline fibrosarcoma cell lines by increasing intracellular drug concentration [45]. The current study emphasizes that the mechanisms-derived information on the 40 nm PEG-AuNP identified a separate but still complementary action on mitochondrial fatty acid β-oxidation, TCA cycle and respiratory chain, drug efflux and uptake transporters, as well as CYP3A4 activity in C3A cells (Fig. 7). To the end, this will highlight AuNP interaction with key biological processes and its underlying molecular mechanism in HCC, which may be further implicated in the development of more effective therapeutic target in HCC treatment.

To validate gene expression analysis from RT^2^ array, the nine genes were selected for real-time PCR. In Additional file 1: Table S1, all nine genes were modulated at LC_50_ of the 40 nm PEG-AuNP. These transcriptional changes were consistent with those in gene expression analysis with PCR arrays (Fig. 6, Additional file 1: Tables S2–S7).

## Conclusions

We have presented that cationic BPEI-, anionic LA-, or neutral PEG-AuNP interaction with human plasma protein corona (PC) caused the changes in *D*_H_, PDI, and z-potential of AuNP and further influenced cellular responses in C3A cells. All bare (no PC) 40 and 80 nm AuNP were cytotoxic to C3A cells besides the 80 nm LA-AuNP but PC completely ameliorated their cytotoxicities besides the 80 nm BPEI-AuNP. The 40 nm bare BPEI-AuNP showed the highest cellular uptake followed by the 40 nm PEG-AuNP and then the 40 nm LA-AuNP, whereas PC suppressed AuNP uptake besides the 80 nm PEG-AuNP. The 40 nm BPEI-AuNP caused biphasic responses of oxidative stress (pro- and antioxidant) in C3A cells, whereas the 40 nm PEG-AuNP was antioxidant. CYP3A4 activity was extensively suppressed by all bare AuNP, irrespective of size and surface charges, whereas PC substantially ameliorated its inhibitory effect on enzyme activity besides the 40 and 80 nm PEG-AuNP. Differentially expressed genes at LC_50_ value of 40 nm PEG-AuNP were mainly involved in mitochondrial fatty acid β-oxidation and to a lesser degree hepatic efflux/uptake transporters. The 40 nm PEG-AuNP inhibited three main enzymes in β-oxidation (acyl-CoA dehydrogenase, enoyl-CoA hydratase, and ketoacyl-CoA thiolase), other enzymes in TCA cycle, and the mitochondrial respiratory chain for ATP production. The 40 nm PEG-AuNP increased the expression of pro-apoptotic genes and decreased anti-apoptotic genes at LC_50_ value. A high level of antioxidants and a low level of pro-oxidants genes were observed in C3A cells exposed to 40 nm PEG-AuNP. In addition, genes of drug efflux and uptake transporters located in both basolateral and canalicular membrane were substantially modulated.

## Additional file

Additional file 1: **Figure S1.** Bare and PC on AuNP surface charge- and size-dependent surface plasmon resonance in complete EMEM* as indicated by UV-Vis spectra at 0 h at 37°. Bare, no protein corona; PC, human plasma protein corona; *, Eagle’s Minimum Essential Medium (EMEM) supplemented with 10% FBS (ATCC^®^, Manassas, VA). **Table S1.** Primer Sequences and Analysis of Gene Expression in C3A cells exposed to the 40 nm Bare PEG-AuNP at Median Lethal Concentrations (LC_50_). **Table S2.** Gene Expression in Mitochondrial Fatty Acid β-oxidation and Mitochondrial Energy Metabolism in C3A Cells after 24 h Exposure to a Median Lethal Concentration (LC_50_) of the 40 nm PEG-AuNP. **Table S3.** Gene Expression in Apoptosis, DNA Damage & Repair, and Necrosis in C3A Cells after 24 h Exposure to a Median Lethal Concentration (LC_50_) of the 40 nm PEG-AuNP. **Table S4.** Gene Expression in ER Stress & Unfolded Protein Response, and Heat Shock Response in C3A Cells after 24 h Exposure to a Median Lethal Concentration (LC_50_) of the 40 nm PEG-AuNP. **Table S5.** Gene Expression in Necrosis, Oxidative Stress & Antioxidant Response and Phase I Metabolism in C3A Cells after 24 h Exposure to a Median Lethal Concentration (LC_50_) of the 40 nm PEG-AuNP. **Table S6.** Gene Expression in Cholestasis, Phospholipidosis & Steatosis in C3A Cells after 24 h Exposure to a Median Lethal Concentration (LC_50_) of the 40 nm PEG-AuNP. **Table S7.** Gene Expression in Drug Efflux and Uptake Transporters in C3A after 24 h Exposure to a Median Lethal Concentrations of the 40 nm PEG-AuNP. (DOCX 168 kb)

## Abbreviations

ANOVA: One-way analysis of variance
AuNP: Gold nanoparticles bare: no PC
BPEI: Branched polyethylenimine
CYP: Cytochrome P450
*D*_H_: Hydrodynamic diameters
DLS: Dynamic light scattering
EDTA: Ethylenediaminetetraacetic acid
EMEM: Eagle’s minimum essential medium
HCC: Human hepatocellular carcinoma
HPTC: Human renal proximal tubular cells
HSD: Tukey’s honest significant difference test
HUVEC: Human umbilical vein cells
ICP-MS: Inductively coupled plasma mass spectrometry
LA: Anionic lipoic acid
LC_50_: Median lethal concentration
MDR: Multidrug resistance
NP: Nanoparticles
PBS: Phosphate-buffered saline
PC: Human plasma protein corona
PDI: Polydispersity index
PEG: Neutral polyethylene glycol
RNAi: RNA interference
RNS: Reactive nitrogen species
ROS: Reactive oxygen species
SO: Superoxide
TEM: Transmission electron microscopy
TFF: Tangential flow filtration
