# Supplementary material for: Assessment of Gold Nanoparticles-Inhibited Cytochrome P450 3A4 Activity and Molecular Mechanisms Underlying Its Cellular Toxicity in Human Hepatocellular Carcinoma Cell Line C3A
Source: Nanoscale Res Lett. 2018 Sep 10;13:279. doi: 10.1186/s11671-018-2684-1 (PMC6134879; doi:10.1186/s11671-018-2684-1)
Supplement: Supplementary file 1 — Figure S1. Bare and PC on AuNP surface charge- and size-dependent surface plasmon resonance in complete EMEM* as indicated by UV-Vis spectra at 0 h at 37°. Bare, no protein corona; PC, human plasma protein corona; *, Eagle’s Minimum Essential Medium (EMEM) supplemented with 10% FBS (ATCC®, Manassas, VA). Table S1. Primer Sequences and Analysis of Gene Expression in C3A cells exposed to the 40 nm Bare PEG-AuNP at Median Lethal Concentrations (LC50). Table S2. Gene Expression in Mitochondrial Fatty Acid β-oxidation and Mitochondrial Energy Metabolism in C3A Cells after 24 h Exposure to a Median Lethal Concentration (LC50) of the 40 nm PEG-AuNP. Table S3. Gene Expression in Apoptosis, DNA Damage & Repair, and Necrosis in C3A Cells after 24 h Exposure to a Median Lethal Concentration (LC50) of the 40 nm PEG-AuNP. Table S4. Gene Expression in ER Stress & Unfolded Protein Response, and Heat Shock Response in C3A Cells after 24 h Exposure to a Median Lethal Concentration (LC50) of the 40 nm PEG-AuNP. Table S5. Gene Expression in Necrosis, Oxidative Stress & Antioxidant Response and Phase I Metabolism in C3A Cells after 24 h Exposure to a Median Lethal Concentration (LC50) of the 40 nm PEG-AuNP. Table S6. Gene Expression in Cholestasis, Phospholipidosis & Steatosis in C3A Cells after 24 h Exposure to a Median Lethal Concentration (LC50) of the 40 nm PEG-AuNP. Table S7. Gene Expression in Drug Efflux and Uptake Transporters in C3A after 24 h Exposure to a Median Lethal Concentrations of the 40 nm PEG-AuNP. (DOCX 168 kb) [file 11671_2018_2684_MOESM1_ESM.docx]

**Additional file 1**

**Assessment of Gold Nanoparticles-Inhibited Cytochrome P450 3A4 Activity and Molecular Mechanisms Underlying its Cellular Toxicity in Human Hepatocellular Carcinoma Cell Line C3A**

Kyoungju Choi* and Hyun Joo

Department of Anatomy & Physiology, Nanotechnology Innovation Center of Kansas State (NICKS), Kansas State University, Manhattan KS, USA

*Corresponding author: Kyoungju Choi

Dr. Kyoungju Choi, Ph.D.

E-mail: [kjchoi@ksu.edu](mailto:kjchoi@ksu.edu)

Dr. Hyun Joo, Ph.D.

E-mail: [hjoo@ksu.edu](mailto:hjoo@ksu.edu)

Fax: 785-532-4953

Table of Contents

**Additional Methods**

Human plasma protein corona preparation

Cell culture and viability measurement

Cellular uptake measurement with inductively coupled plasma mass spectrometry (ICP-MS)

Oxidative/nitrosative stress measurements

Gene expression profiling (Real time PCR and quantification)

**Figure S1**

**Table S1**

**Table S2**

**Table S3**

**Table S4**

**Table S5**

**Table S6**

**Table S7**

**References**

**Additional Methods**

*Human Plasma Protein Corona Preparation*

Pooled human blood plasma of healthy donors (n=5) were obtained from the Biological Specialty Corp. (Colmar, PA). The handling of whole blood was conducted at the Biological Specialty Corp. with an approval by Food and Drug Administration (FDA), Department of Health and Human Services Public Health Service, Blood establishment registration and product listing (Registration number of FEI, 3002965662 and CFN, 2531847 and US license number of 865). An exclusion criteria included the samples from donors with blood-borne infectious diseases and blood disorders (Hepatitis A or B, HIV). Human blood plasma samples were frozen on arrival at Kansas State University (KSU). Upon arrival, it was thawed on ice, aliquoted and then stored at -80°C until further use. Human plasma samples were handled under the biological safety cabinets at BSL2 level laboratory, Dept. of Anatomy & Physiology, KSU. AuNP was incubated with human plasma at physiological plasma volume in total blood volume (55%, v/v) in an orbital shaking/rotating incubator at 37°C for 1h. The unbound and weakly associated proteins around AuNP were removed by centrifugation at 20,000xg for 20 min followed by washing in PBS. The final protein corona (PC) coated AuNP was dispersed in PBS and diluted in cell culture medium that consisted of Eagle’s Minimum Essential Medium (EMEM) (ATCC^®^, Manassas, VA) supplemented with 10% fetal bovine serum (FBS) for further physicochemical characterization and/or dosing.

*Cell culture and viability measurement*

Human hepatocellular carcinoma C3A cells (ATCC^®^CRL-10741™) were purchased from ATCC^®^ (Manassas, VA), cultured in complete Eagle’s Minimum Essential Medium (EMEM) (ATCC^®^, Manassas, VA) supplemented with 10% FBS and expanded to approximately 80% confluence in T75 flask with medium changes every 4d. After 0.25% (w/v) trypsin-0.53mM ethylenediaminetetraacetic acid (EDTA) digestion, cells were plated in 96-well plates at 8 x 10^4^ cells per well and incubated at 37°C in a humidified atmosphere of 95% air and 5% CO_2._ After 48h incubation, cells were dosed with AuNP in the absence and presence of PC. The C3A cells between passage 9 and 12 were used for the dosing.

The C3A viability was determined using the alamarBlue^®^ viability assay (Thermo Sci., Waltham, MA) as described [1,2]. Cells in the 96-well plates were treated with the 40 and 80nm BPEI-, LA- and PEG-AuNP with and without PC ranging from 0 to 250µg/cm^2^. After 24h, 10% of alamarBlue^®^ reagent in complete EMEM (v/v) was added to the cell culture and incubated for 3h at 37°C. The complete EMEM served as a dispersant. Fluorescence, proportional to cell viability was quantified with a microplate reader and then normalized by subtracting the background fluorescence. The cell viability was expressed as a percentage relative to control cell group.

*Cellular uptake measurement with inductively coupled plasma mass spectrometry (ICP-MS)*

Time-dependent cellular uptake of AuNP in C3A cells was determined as previously described [1,3]. Preliminary study of optimal cell density and concentrations of AuNP showed that cell uptake was linear from 8x10^4^ cells to 1.25x10^6^ cells ranging from 0.31µg/cm^2^ to 15.63µg/cm^2^. C3A cells in 96-well plates (8 x 10^4^ cells per well) were dosed with 40 and 80nm BPEI-, LA- and PEG-AuNP with and without PC at 1.56μg/cm^2^ and collected at 0.5, 1, 3, 6, 12 and 24h. The methods for a removal of the cell surface-binding AuNP and its non-specific binding to 96-well plate with an etching agent (iodine/potassium iodine aqueous solution), cell harvest and digestion in aqua regia have been described in extensive detail in a recent publication [4]. For a quantification of intracellular Au concentration, a sample was diluted in 2% HCl/1% HNO_3_ aqueous solution and subject to the NexION^™^ 350X inductively coupled plasma mass spectrometer (ICP-MS) (PerkinElmer, Waltham, MA). AuNP uptake in the C3A cells was calculated as previously reported and expressed as the number of AuNP per cell [5]. A five-point calibration curve (1ng/mL to 100ng/mL) was used. Three independent experiments were conducted, and results were expressed as number of AuNP (N_Au_) per hepatocyte calculated using equation (1) with the following modification [1,5].

| N_Au_ | = | TA_Au_ | = | 6 x V_S_ x M x N x m | = | 6 x V_S_ x M x m | Equation (1) |
| --- | --- | --- | --- | --- | --- | --- | --- |
|  |  | *an*_Au_ |  | D^3^ x π x N x ρ |  | D^3^ x π x ρ |  |

where a total atom number of AuNP in sample (TA_Au_) is TA_Au_ = V_S_ x M x N, with V_S_ = sample volume (0.1 mL), M = molarity of AuNP (mol/L) and N = Avogadro’s number, 6.022x10^23^ atoms/mol

Atom number per AuNP (*an*_NP_) is

| *an*_Au_ | = | V_Au_ x N x ρ |
| --- | --- | --- |
|  |  | m |

Where volume of AuNP (V_Au_) is: V_Au =_ 1/6 x D^3^ x π with D = diameter of AuNP (40 or 80nm), m = molar mass of Au (197 g/mol) and ρ = a density of cubic Au (19.3g/cm^3^).

*Oxidative/Nitrosative Stress Measurements*

Total oxygen/nitrosative stress in C3A cells exposed to the 40 and 80nm BPEI-AuNP and PEG-AuNP with and without PC were determined as previously reported [6]. Total reactive oxygen species (ROS)/superoxide (SO) detection kit (Enzo Life Sciences, Farmingdale, NY) was used to detect ROS, reactive nitrogen species (RNS) and SO. Cells in 96-well plates were dosed with AuNP up to 125µg/cm^2^ and subsequently incubated with a mixture of green dye (ROS/RNS detection) and orange dye (SO detection) in washing buffer (a 1:2500 dilution, v/v). Fluorescence, proportional to the increase in ROS/RNS (Ex488/Em520 nm) or SO (Ex550/Em610 nm) was measured with a microplate reader at 0, 1, 3 and 24h, normalized to controls, and plotted as a fold change compared to the control cell group. Controls were assigned to assess the interaction of AuNP with the detection dyes. The pyocyanin was used as a ROS inducer and served as a positive control.

*Gene expression profiling (Real time PCR and quantification)*

The 1 µg of total RNA extracted from C3A cells with an average RNA integrity numbers (RIN) of 7.8 was subject to First strand cDNA synthesis using RT^2^First Strand kit (Qiagen Inc., Valencia, CA). The cDNA was mixed with RT^2^ SYBR green mastermix and applied to the Human Molecular Toxicology Pathway Finder and Human Drug Transporters RT^2^ Profiler™ PCR Array in the QuantStudio^™^ 7 Flex.The Human Molecular Toxicology Pathway Finder RT^2^ Profiler^™^ PCR arrays profiles the expression of 370 genes related to 13 different functional pathways. Human drug transporters PCR array was used to characterize AuNP-relevant transporters with a total of 29 genes of human ATP-binding cassette (ABC) transporters, 55 genes of the solute carrier (SLC) transporters and 9 genes of other transporters. A total of five different housekeeping genes (glyceraldehyde-3-phosphate dehydrogenase, β-2-microglobulin, ribosomal protein large P0, and hypoxanthine phosphoribosyltransferase 1 and β-actin) were used. The real time PCR cycling was 95°C for 10min and 40 cycles of denaturation at 95°C for 15sec, amplification at 55°C for 40 sec, and annealing at 72°C for 30 sec. Cycle threshold (Ct) from QuantStudio Real time PCR software v1.1 was used to determine the changes in gene of interest (GOI) with the Webportal

(<http://www.qiagen.com/us/shop/genes-and-pathways/data-analysis-center-overview-page/>). Briefly, the ΔCt for each GOI was calculated with the Ct values of GOI and housekeeping gene and averaged for each gene across replicate arrays for control group and treated group. An expression level of GOI was calculated as 2^-average ΔCt^_._ Differential gene expression was expressed as fold change of expression in the treatment group with 40nm bare PEG-AuNP relative to the control group.

To validate the RT^2^ PCR array data, the expression of the nine genes was selected and subject to qPCR. The cDNA synthesis and real-time PCR were performed with QuantiNoa™ Reverse Transcription kit and SYBR®Green PCR kit, respectively. Primer sequences are summarized in Supplementary Table S1. All PCR reactions were conducted in triplicate.


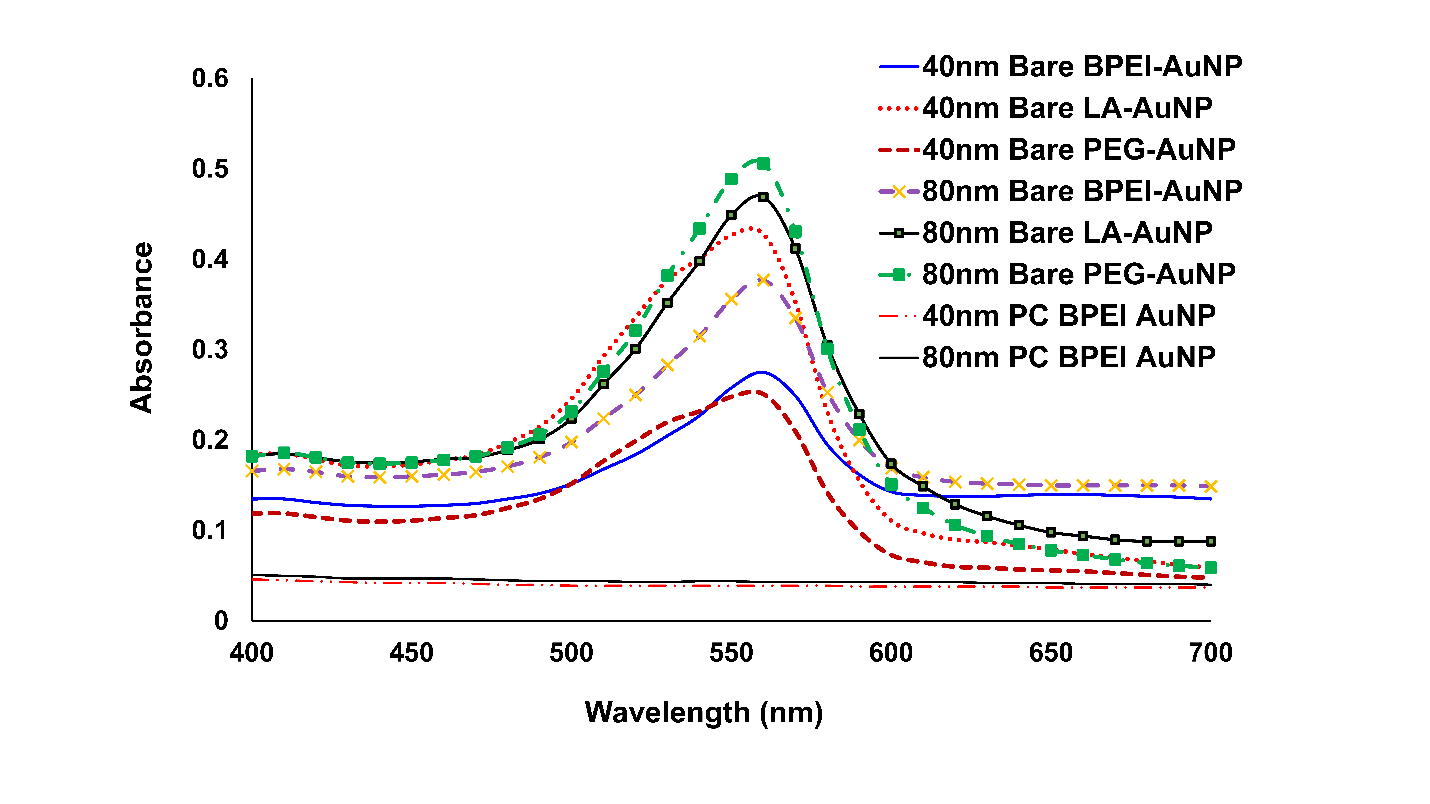


**Figure S1**. Bare and PC on AuNP surface charge- and size-dependent surface plasmon resonance in complete EMEM* as indicated by UV-Vis spectra at 0h at 37°. Bare, no protein corona; PC, human plasma protein corona; *, Eagle’s Minimum Essential Medium (EMEM) supplemented with 10% FBS (ATCC^®^, Manassas, VA).

**Table S1**. Primer Sequences and Analysis of Gene Expression in C3A Cells Exposed to the 40nm Bare PEG-AuNP at a Median Lethal Concentrations (LC_50_).

| Gene symbol | GenBank | Primer Sequence (5’→ 3’) | Amplicon size | Fold change |
| --- | --- | --- | --- | --- |
| ABCB4 | NM_000443 | SENSE 5’-TGTCTCAGGAGCCTATCCTATT-3’ | 101 bp | -12.2±0.1 |
|  |  | ANTISENSE 5’-GGCTGCACTCACAATTTCATC-3’ |  |  |
| ALDH1A1 | NM_000689 | SENSE 5’-GTCAAACCAGCAGAGCAAAC-3’ | 107 bp | -5.8±0.2 |
|  |  | ANTISENSE 5’-GGCCCATAACCAGGAACAATA-3’ |  |  |
| CYP3A4 | NM_017460 | SENSE 5’-CCAAATCAGTGTGAGGAGGTAG-3’ | 96 bp | -9.2±0.2 |
|  |  | ANTISENSE 5’- CAGAGCTCAGGAGGAGTTAATG-3’ |  |  |
| DDIT3 | NM_004083 | SENSE 5’-CAAGAGGTCCTGTCTTCAGATG -3’ | 95 bp | 5.5±0.1 |
|  |  | ANTISENSE 5’-GGGTCAAGAGTGGTGAAGATT-3’ |  |  |
| ERCC6 | NM_000124 | SENSE 5’-GTTGACGTGGAGAAGGAGTAT-3’ | 101 bp | -16.5±0.2 |
|  |  | ANTISENSE 5’-TGAGGGCTAAGCTGTTCAATAA-3’ |  |  |
| SLC51A | NM_152672 | SENSE 5’-GCTTGTTCGCCTCCCTATT-3’ | 96 bp | -17.2±0.1 |
|  |  | ANTISENSE 5’-TCGTGTCAGCACAGTCATTAG- 3’ |  |  |
| GPX2 | NM_002083 | SENSE 5’-AGAATGATGGCACCTTCCTAAA -3’ | 101 bp | 6.2±0.2 |
|  |  | ANTISENSE 5’-TCCACACCTGCCCTTTATTG-3’ |  |  |
| IL1B | NM_000576 | SENSE 5’-TGATGGCTTATTACAGTGGCAATG-3’ | 140 bp | -9.4±0.2 |
|  |  | ANTISENSE 5’-GTAGTGGTGGTCGGAGATTCG-3’ |  |  |
| ICAM1 | NM_000201 | SENSE 5’-CCCTGATGGGCAGTCAACAG-3’ | 119 bp | -5.3±0.1 |
|  |  | ANTISENSE 5’-GGCAGCGTAGGGTAAGGTTC-3’ |  |  |
| GAPDH | NM_002046 | SENSE 5’-CAAGAGCACAAGAGGAAGAGAG-3’ | 102 bp |  |
|  |  | ANTISENSE 5’-CTACATGGCAACTGTGAGGAG-3’ |  |  |

Bare, No Protein Corona

**Table S2.** Gene Expression in Mitochondrial Fatty Acid β-oxidation and Mitochondrial Energy Metabolism in C3A Cells after 24h Exposure to a Median Lethal Concentration (LC_50_) of the 40nm PEG-AuNP.

| **Symbol** | **GenBank** | **Fold changes** | **Description** |
| --- | --- | --- | --- |
| **Mitochondrial Fatty Acid β-Oxidation** | | | |
| ACAA1 | NM_001607 | -7.3±0.1 | Acetyl-CoA acyltransferase 1 |
| ACAA2 | NM_006111 | -14.9±0.9 | Acetyl-CoA acyltransferase 2 |
| ACAD11 | NM_032169 | -8.0±1.1 | Acyl-CoA dehydrogenase family, member 11 |
| ACAD9 | NM_014049 | -3.7±0.3 | Acyl-CoA dehydrogenase family, member 9 |
| ACADM | NM_000016 | -13.6±0.1 | Acyl-CoA dehydrogenase, C-4 to C-12 straight chain |
| ACADS | NM_000017 | -1.9±0.4 | Acyl-CoA dehydrogenase, C-2 to C-3 short chain |
| ACADSB | NM_001609 | -8.8±0.8 | Acyl-CoA dehydrogenase, short/branched chain |
| ACAT1 | NM_000019 | -16.0±0.6 | Acetyl-CoA acetyltransferase 1 |
| ACOT1 | NM_001037161 | -4.1±0.8 | Acyl-CoA thioesterase 1 |
| ACOT6 | NM_001037162 | -2.2±0.8 | Acyl-CoA thioesterase 6 |
| ACOT7 | NM_181866 | -6.4±0.1 | Acyl-CoA thioesterase 7 |
| ACOT8 | NM_005469 | -4.9±0.1 | Acyl-CoA thioesterase 8 |
| ACOT9 | NM_001033583 | -10.0±0.1 | Acyl-CoA thioesterase 9 |
| ACOX1 | NM_004035 | -10.1±1.2 | Acyl-CoA oxidase 1, palmitoyl |
| ACOX2 | NM_003500 | -13.5±0.9 | Acyl-CoA oxidase 2, branched chain |
| ACOX3 | NM_003501 | -7.8±0.5 | Acyl-CoA oxidase 3, pristanoyl |
| BDH2 | NM_020139 | -6.2±0.6 | 3-hydroxybutyrate dehydrogenase, type 2 |
| CPT1A | NM_001876 | -13.0±0.6 | Carnitine palmitoyltransferase 1A (liver) |
| CPT2 | NM_000098 | -12.4±1.5 | Carnitine palmitoyltransferase 2 |
| CRAT | NM_000755 | -4.9±0.1 | Carnitine O-acetyltransferase |
| CROT | NM_021151 | -4.1±0.4 | Carnitine O-octanoyltransferase |
| DECR1 | NM_001359 | -12.3±1.7 | 2,4-dienoyl CoA reductase 1, mitochondrial |
| ECHS1 | NM_004092 | -12.3±1.1 | Enoyl CoA hydratase, short chain, 1, mitochondrial |
| EHHADH | NM_001966 | -10.7±0.6 | Enoyl-CoA, hydratase/3-hydroxyacyl CoA dehydrogenase |
| GCDH | NM_000159 | -4.5±0.7 | Glutaryl-CoA dehydrogenase |
| HADHA | NM_000182 | -18.9±1.0 | Enoyl-CoA hydratase, α subunit |
| **Mitochondrial Energy Metabolism (Tricarboxylic Acid, TCA Cycle)** | | | |
| ACLY | NM_001096 | -14.9±1.8 | ATP citrate lyase |
| ACO2 | NM_001098 | -2.9±0.5 | Aconitase 2, mitochondrial |
| COX6B1 | NM_001863 | -2.7±0.1 | Cytochrome c oxidase subunit Vib polypeptide 1 (ubiquitous) |
| CS | NM_004077 | -12.1±1.5 | Citrate synthase |
| CYC1 | NM_001916 | -8.4±0.2 | Cytochrome c-1 |
| DLD | NM_000108 | -15.7±3.0 | Dihydrolipoamide dehydrogenase |
| FH | NM_000143 | -9.1±0.5 | Fumarate hydratase |
| IDH2 | NM_002168 | -16.9±0.1 | Isocitrate dehydrogenase 2 (NADP+), mitochondrial |
| IDH3A | NM_005530 | -6.1±0.1 | Isocitrate dehydrogenase 3 (NAD+) alpha |
| IDH3B | NM_174856 | -16.9±1.1 | Isocitrate dehydrogenase 3 (NAD+) beta |
| IDH3G | NM_174869 | -5.6±0.3 | Isocitrate dehydrogenase 3 (NAD+) gamma |
| MDH2 | NM_005918 | -8.5±0.1 | Malate dehydrogenase 2, NAD (mitochondrial) |
| OGDH | NM_002541 | -4.9±0.3 | Oxoglutarate (alpha-ketoglutarate) dehydrogenase |
| SDHA | NM_004168 | -6.4±0.7 | Succinate dehydrogenase complex, subunit A, flavoprotein |
| SDHB | NM_003000 | -10.3±0.3 | Succinate dehydrogenase complex, subunit B, iron sulfur |
| SDHD | NM_003002 | -17.3±0.3 | Succinate dehydrogenase complex, subunit D |
| SUCLA2 | NM_003850 | -15.5±5.0 | Succinate-CoA ligase, ADP-forming, β subunit |
| SUCLG1 | NM_003849 | -14.5±2.4 | Succinate-CoA ligase, α subunit |
| SUCLG2 | NM_003848 | -6.9±0.6 | Succinate-CoA ligase, GDP-forming, β subunit |
| UCP1 | NM_021833 | -5.1±0.8 | Uncoupling protein 1 (mitochondrial, proton carrier) |
| UCP2 | NM_003355 | -5.1±1.2 | Uncoupling protein 2 (mitochondrial, proton carrier) |

**Table S3**. Gene Expression in Apoptosis, DNA Damage & Repair, and Necrosis in C3A Cells after 24h Exposure to a Median Lethal Concentration (LC_50_) of the 40nm PEG-AuNP.

| **Symbol** | **GenBank** | **Fold changes** | **Description** |
| --- | --- | --- | --- |
| **Apoptosis** | | | |
| ABL1 | NM_005157 | -6.8±0.6 | C-abl oncogene 1, non-receptor tyrosine kinase |
| AKT1 | NM_005163 | -3.2±0.3 | AKT serine/threonine kinase |
| ALB | NM_000477 | -20.6±5.0 | Albumin |
| APAF1 | NM_001160 | -4.9±1.4 | Apoptotic peptidase activating factor 1 |
| BAD | NM_004322 | -4.1±0.1 | BCL2-associated agonist of cell death |
| BAK1 | NM_001188 | -3.4±0.1 | BCL2-antagonist/killer 1 |
| BAX | NM_004324 | -4.0±0.2 | BCL2-associated X protein |
| BCL2 | NM_000633 | -3.1±0.7 | B-cell lymphoma 2 |
| BCL2L1 | NM_138578 | -9.8±1.2 | BCL2-like 1 |
| BID | NM_001196 | -7.4±0.1 | BH3 interacting domain death agonist |
| CASP3 | NM_004346 | 18.4±2.8 | Caspase 3, apoptosis-related cysteine peptidase |
| CASP7 | NM_001227 | 9.1±0.1 | Caspase 7, apoptosis-related cysteine peptidase |
| CASP9 | NM_001229 | 5.4±0.2 | Caspase 9, apoptosis-related cysteine peptidase |
| CFLAR | NM_003879 | -7.0±0.2 | CASP8 and FADD-like apoptosis regulator |
| FADD | NM_003824 | -5.0±0.4 | Fas (TNFRSF6)-associated via death domain |
| FAS | NM_000043 | -9.1±0.8 | Fas (TNF receptor superfamily, member 6) |
| GADD45A | NM_001924 | -3.0±0.1 | Growth arrest and DNA-damage-inducible, alpha |
| MCL1 | NM_021960 | -11.3±0.3 | Myeloid leukemia cell differentiation protein (BCL2 family) |
| TNFRSF10A | NM_003844 | 5.8±0.6 | Tumor necrosis factor receptor superfamily, member 10a |
| TNFRSF10B | NM_003842 | 9.3±0.7 | Tumor necrosis factor receptor superfamily, member 10b |
| TNFRSF1A | NM_001065 | -3.9±0.7 | Tumor necrosis factor receptor superfamily, member 1A |
| TNFSF10 | NM_003810 | 10.6±1.0 | Tumor necrosis factor (ligand) superfamily, member 10 |
| TP53 | NM_000546 | -7.1±0.2 | Tumor protein p53 |
| XIAP | NM_001167 | -7.6±0.7 | X-linked inhibitor of apoptosis |
| **DNA Damage & Repair** | | | |
| APEX1 | NM_080649 | -12.6±0.2 | APEX nuclease (multifunctional DNA repair enzyme) 1 |
| BRCA1 | NM_007294 | -8.4±1.8 | Breast cancer 1, early onset |
| BRCA2 | NM_000059 | -11.4±1.9 | Breast cancer 2, early onset |
| CDKN1A | NM_000389 | -3.1±0.1 | Cyclin-dependent kinase inhibitor 1A (p21, Cip1) |
| CHEK1 | NM_001274 | 10.4±1.3 | CHK1 checkpoint homolog |
| CHEK2 | NM_007194 | 12.2±0.3 | CHK2 checkpoint homolog |
| CLEC18A | NM_182619 | -2.5±0.1 | C-type lectin domain family 18, member A |
| ERCC1 | NM_001983 | 6.8±0.4 | Excision repair cross-complementing group 1 |
| ERCC2 | NM_000400 | 3.6±1.4 | Excision repair cross-complementing group 2 |
| ERCC3 | NM_000122 | 9.9±0.1 | Excision repair cross-complementing group 3 |
| ERCC5 | NM_000123 | -10.7±0.3 | Excision repair cross-complementing group 5 |
| ERCC6 | NM_000124 | -16.6±5.8 | Excision repair cross-complementing group 6 |
| LIG4 | NM_002312 | 18.0±0.2 | Ligase IV, DNA, ATP-dependent |
| MDM2 | NM_002392 | -2.7±0.1 | Mdm2 p53 binding protein homolog |
| MGMT | NM_002412 | -2.3±0.1 | O-6-methylguanine-DNA methyltransferase |
| MLH1 | NM_000249 | -9.3±1.2 | MutL homolog 1, colon cancer, nonpolyposis type 2 |
| OGG1 | NM_002542 | -7.8±0.3 | 8-oxoguanine DNA glycosylase |
| PARP1 | NM_001618 | -13.8±1.0 | Poly (ADP-ribose) polymerase 1 |
| PCNA | NM_182649 | -10.2±0.7 | Proliferating cell nuclear antigen |
| PRKDC | NM_006904 | -19.4±0.2 | Protein kinase, DNA-activated, catalytic polypeptide |
| RAD51 | NM_002875 | -7.9±1.1 | RAD51 homolog |
| XPA | NM_000380 | -8.1±1.3 | Xeroderma pigmentosum, complementation group A |
| XRCC1 | NM_006297 | -3.5±0.3 | X-ray repair complementing defective repair 1 |
| XRCC5 | NM_021141 | -5.1±0.3 | X-ray repair complementing defective repair 5 |

**Table S4.** Gene Expression in ER Stress & Unfolded Protein Response, and Heat Shock Response in C3A Cells after 24h Exposure to a Median Lethal Concentration (LC_50_) of the 40nm PEG-AuNP.

| **Symbol** | **GenBank** | **Fold changes** | **Description** |
| --- | --- | --- | --- |
| **ER Stress & Unfolded Protein Response** | | | |
| AMFR | NM_001144 | -9.4±1.2 | Autocrine motility factor receptor |
| ATF4 | NM_001675 | -9.9±0.2 | Activating transcription factor 4 |
| ATF6 | NM_007348 | -20.2±4.0 | Activating transcription factor 6 |
| DDIT3 | NM_004083 | 3.5±0.2 | DNA-damage-inducible transcript 3 |
| DERL1 | NM_024295 | -10.5±0.4 | Der1-like domain family, member 1 |
| EDEM1 | NM_014674 | -6.7±0.1 | ER degradation enhancer, mannosidase alpha-like 1 |
| EIF2AK3 | NM_004836 | -16.9±2.3 | Eukaryotic translation initiation factor 2-alpha kinase 3 |
| FBXO6 | NM_018438 | -14.6±4.0 | F-box protein 6 |
| HTRA2 | NM_013247 | -12.4±2.9 | HtrA serine peptidase 2 |
| MBTPS1 | NM_003791 | -3.2±0.1 | Membrane-bound transcription factor peptidase, site 1 |
| NUCB1 | NM_006184 | -10.5±0.1 | Nucleobindin 1 |
| OS9 | NM_006812 | -9.1±0.5 | Osteosarcoma amplified 9, endoplasmic reticulum lectin |
| PFDN5 | NM_002624 | -4.6±0.1 | Prefoldin subunit 5 |
| SYVN1 | NM_172230 | -4.7±0.6 | Synovial apoptosis inhibitor 1, synoviolin |
| UBE2G2 | NM_182688 | 3.9±0.3 | Ubiquitin-conjugating enzyme E2G 2 |
| UBE2J2 | NM_194458 | 3.7±0.6 | Ubiquitin-conjugating enzyme E2, J2 |
| UBXN4 | NM_014607 | -13.3±1.6 | UBX domain protein 4 |
| VCP | NM_007126 | -13.3±0.3 | Valosin containing protein |
| XBP1 | NM_005080 | -5.9±0.7 | X-box binding protein 1 |
| VIMP | NM_203472 | -6.3±0.4 | Selenoprotein S |
| **Heat Shock Response** | | | |
| DNAJA1 | NM_001539 | -9.1±0.4 | DnaJ (Hsp40) homolog, subfamily A, member 1 |
| DNAJA2 | NM_005880 | -8.9±0.2 | DnaJ (Hsp40) homolog, subfamily A, member 2 |
| DNAJA3 | NM_005147 | -4.5±0.1 | DnaJ (Hsp40) homolog, subfamily A, member 3 |
| DNAJB1 | NM_006145 | -2.9±0.3 | DnaJ (Hsp40) homolog, subfamily B, member 1 |
| DNAJB6 | NM_005494 | -5.6±0.1 | DnaJ (Hsp40) homolog, subfamily B, member 6 |
| DNAJC3 | NM_006260 | -8.7±0.9 | DnaJ (Hsp40) homolog, subfamily C, member 3 |
| DNAJC5 | NM_025219 | -3.4±0.6 | DnaJ (Hsp40) homolog, subfamily C, member 5 |
| HSF1 | NM_005526 | -4.6±0.3 | Heat shock transcription factor 1 |
| HSF2 | NM_004506 | -14.7±0.3 | Heat shock transcription factor 2 |
| HSP90AA1 | NM_001017963 | -4.1±0.1 | Heat shock protein 90kDa alpha (cytosolic), class A member 1 |
| HSP90AB1 | NM_007355 | -9.8±0.2 | Heat shock protein 90kDa alpha (cytosolic), class B member 1 |
| HSP90B1 | NM_003299 | -15.5±0.7 | Heat shock protein 90kDa beta (Grp94), member 1 |
| HSPA1A | NM_005345 | 10.2±0.4 | Heat shock 70kDa protein 1A |
| HSPA1B | NM_005346 | 14.2±0.6 | Heat shock 70kDa protein 1B |
| HSPA1L | NM_005527 | -2.2±0.1 | Heat shock 70kDa protein 1-like |
| HSPA4 | NM_002154 | -11.1±0.1 | Heat shock 70kDa protein 4 |
| HSPA5 | NM_005347 | -6.6±1.0 | Heat shock 70kDa protein 5 (glucose-regulated protein, 78kDa) |
| HSPA8 | NM_006597 | -9.8±0.4 | Heat shock 70kDa protein 8 |
| HSPA9 | NM_004134 | -9.4±0.1 | Heat shock 70kDa protein 9, mortalin |
| HSPB1 | NM_001540 | -2.0±0.2 | Heat shock 27kDa protein 1 |
| HSPBAP1 | NM_024610 | -9.9±1.3 | HSPB (heat shock 27kDa) associated protein 1 |
| HSPD1 | NM_002156 | -16.1±0.4 | Heat shock 60kDa protein 1 (chaperonin) |
| HSPE1 | NM_002157 | -4.6±0.3 | Heat shock 10kDa protein 1 (chaperonin 10) |
| HSPH1 | NM_006644 | -3.2±0.1 | Heat shock 105kDa/110kDa protein 1 |

**Table S5**. Gene Expression in Necrosis, Oxidative Stress & Antioxidant Response and Phase I Metabolism in C3A Cells after 24h Exposure to a Median Lethal Concentration (LC_50_) of the 40nm PEG-AuNP.

| **Symbol** | **GenBank** | **Fold changes** | **Description** |
| --- | --- | --- | --- |
| **Necrosis** | | | |
| ATP6V1G2 | NM_130463 | -2.5±0.2 | ATPase, H+ transporting, lysosomal 13kDa, V1 subunit G2 |
| BMF | NM_033503 | -2.7±0.5 | Bcl2 modifying factor |
| COMMD4 | NM_017828 | -8.8±1.4 | COMM domain containing 4 |
| DEFB1 | NM_005218 | -3.3±0.2 | Defensin, beta 1 |
| GRB2 | NM_002086 | -13.8±0.1 | Growth factor receptor-bound protein 2 |
| NUDT13 | NM_015901 | -9.6±3.2 | Nudix (nucleoside diphosphate linked moiety X) |
| PARP2 | NM_005484 | -11.2±0.1 | Poly (ADP-ribose) polymerase 2 |
| PVR | NM_006505 | -6.1±0.5 | Poliovirus receptor |
| RAB25 | NM_020387 | -16.3±0.5 | RAB25, member RAS oncogene family |
| SPATA2 | NM_006038 | -2.0±0.1 | Spermatogenesis associated 2 |
| TMEM57 | NM_018202 | -10.9±1.3 | Transmembrane protein 57 |
| TNFAIP8L1 | NM_152362 | -3.3±0.4 | Tumor necrosis factor, alpha-induced protein 8-like 1 |
| TXNL4B | NM_017853 | -6.3±0.2 | Thioredoxin-like 4B |
| **Oxidative Stress & Antioxidant Response** | | | |
| CAT | NM_001752 | 18.1±0.1 | Catalase |
| DHCR24 | NM_014762 | -9.1±0.9 | 24-dehydrocholesterol reductase |
| GPX1 | NM_000581 | 9.8±0.1 | Glutathione peroxidase 1 |
| GPX2 | NM_002083 | 3.8±0.1 | Glutathione peroxidase 2 |
| GPX4 | NM_002085 | 4.3±0.1 | Glutathione peroxidase 4 (phospholipid hydroperoxidase) |
| NUDT15 | NM_018283 | -5.2±0.2 | Nudix (nucleoside diphosphate linked moiety X) |
| PPP1R15B | NM_032833 | -17.0±4.0 | Protein phosphatase 1, regulatory (inhibitor) subunit 15B |
| PRDX1 | NM_002574 | 8.4±0.6 | Peroxiredoxin 1 |
| PRDX2 | NM_005809 | 4.6±0.1 | Peroxiredoxin 2 |
| PRDX6 | NM_004905 | 9.0±0.4 | Peroxiredoxin 6 |
| SOD1 | NM_000454 | -8.0±0.4 | Superoxide dismutase 1, soluble |
| TXNIP | NM_006472 | -4.8±0.1 | Thioredoxin interacting protein |
| **Phase I Metabolism** | | | |
| CYP3A4 | NM_017460 | -7.0±0.3 | Cytochrome P450, family 3, subfamily A, polypeptide 4 |
| ESD | NM_001984 | -12.2±0.5 | Esterase D |

**Table S6.** Gene Expression in Cholestasis, Phospholipidosis & Steatosis in C3A Cells after 24h Exposure to a Median Lethal Concentration (LC_50_) of the 40nm PEG-AuNP.

| **Symbol** | **GenBank** | **Fold changes** | **Description** |
| --- | --- | --- | --- |
| **Cholestasis** | | | |
| APOE | NM_000041 | -5.2±2.3 | Apolipoprotein E |
| ATP8B1 | NM_005603 | -15.4±1.7 | ATPase, aminophospholipid transporter, class I, type 8B, member 1 |
| DLAT | NM_001931 | -14.1±0.6 | Dihydrolipoamide S-acetyltransferase |
| ESR1 | NM_000125 | -2.7±0.3 | Estrogen receptor 1 |
| ICAM1 | NM_000201 | -4.0±0.1 | Intercellular adhesion molecule 1 |
| IL1B | NM_000576 | -8.6±1.0 | Interleukin 1, beta |
| JAG1 | NM_000214 | -3.3±0.4 | Jagged 1 |
| NR1H4 | NM_005123 | -10.7±1.8 | Nuclear receptor subfamily 1, group H, member 4 |
| NUP210 | NM_024923 | -4.9±0.1 | Nucleoporin 210kDa |
| RDX | NM_002906 | -12.8±1.1 | Radixin |
| SLC51A | NM_152672 | -16.3±2.0 | Organic solute transporter alpha |
| SLC51B | NM_178859 | -8.9±0.7 | Organic solute transporter beta |
| RDX | NM_002906 | -12.8±1.1 | Radixin |
| TGFB1 | NM_000660 | -3.2±0.1 | Transforming growth factor, beta 1 |
| **Phospholipidosis** | | | |
| ALDH1A1 | NM_000689 | -4.2±0.1 | Aldehyde dehydrogenase 1 family, member A1 |
| ASAH1 | NM_004315 | -13.2±1.0 | N-acylsphingosine amidohydrolase (acid ceramidase) 1 |
| ASNS | NM_183356 | -12.8±1.6 | Asparagine synthetase (glutamine-hydrolyzing) |
| CES2 | NM_198061 | -2.6±0.1 | Carboxylesterase 2 |
| CTSB | NM_001908 | -14.4±0.1 | Cathepsin B |
| EPHX1 | NM_000120 | -9.4±0.1 | Epoxide hydrolase 1, microsomal (xenobiotic) |
| HPN | NM_002151 | -9.8±0.5 | Hepsin |
| INHBE | NM_031479 | -7.6±0.1 | inhibin, beta E |
| LSS | NM_002340 | -9.6±0.1 | Lanosterol synthase (2,3-oxidosqualene-lanosterol cyclase) |
| MANBA | NM_005908 | -23.0±0.8 | Mannosidase, beta A, lysosomal |
| MLX | NM_198205 | -16.2±1.9 | MAX-like protein X |
| MRPS18B | NM_014046 | -12.0±1.3 | Mitochondrial ribosomal protein S18B |
| POR | NM_000941 | -5.7±0.1 | P450 (cytochrome) oxidoreductase |
| SLC2A3 | NM_006931 | -10.9±0.1 | Solute carrier family 2 (facilitated glucose transporter), member 3 |
| SMPD1 | NM_000543 | -7.9±0.3 | Sphingomyelin phosphodiesterase 1, acid lysosomal |
| STBD1 | NM_003943 | -4.6±0.7 | Starch binding domain 1 |
| TAGLN | NM_003186 | -2.0±0.1 | Transgelin |
| TIMM10B | NM_012192 | -7.8±1.2 | Fracture callus 1 homolog |
| WIPI1 | NM_017983 | -13.9±0.2 | WD repeat domain, phosphoinositide interacting 1 |
| **Steatosis** | | | |
| ACACA | NM_198834 | 6.8±0.4 | Acetyl-CoA carboxylase alpha |
| ADK | NM_001123 | -15.1±0.6 | Adenosine kinase |
| ALDH2 | NM_000690 | -9.6±0.6 | Aldehyde dehydrogenase 2 family (mitochondrial) |
| CD36 | NM_000072 | -18.7±2.1 | CD36 molecule (thrombospondin receptor) |
| COMT | NM_000754 | -4.3±0.1 | Catechol-O-methyltransferase |
| ENO1 | NM_001428 | -19.6±1.9 | Enolase 1, (alpha) |
| FASN | NM_004104 | -8.6±0.8 | Fatty acid synthase |
| KHK | NM_000221 | -4.4±0.3 | Ketohexokinase (fructokinase) |
| MAPK8 | NM_002750 | -10.8±1.1 | Mitogen-activated protein kinase 8 |
| PCCA | NM_000282 | -8.4±0.3 | Propionyl CoA carboxylase, alpha polypeptide |
| PNPLA3 | NM_025225 | -13.1±0.1 | Patatin-like phospholipase domain containing 3 |
| PPARA | NM_005036 | 5.2±0.1 | Peroxisome proliferator-activated receptor alpha |
| SREBF1 | NM_004176 | -3.8±0.1 | Sterol regulatory element binding transcription factor 1 |

**Table S7.** Gene Expression in Drug Efflux and Uptake Transporters in C3A after 24h Exposure to a Median Lethal Concentrations of the 40nm PEG-AuNP.

| **Symbol** | **GenBank** | **Fold changes** | **Description** |
| --- | --- | --- | --- |
| **ABC transporters** | | | |
| ABCA1 | NM_005502 | -8.6±0.1 | ATP ­binding cassette, sub­family A (ABC1), member 1, cholesterol efflux regulator protein (CERP) |
| ABCB1 | NM_000927 | -12.4±0.2 | ATP­ binding cassette, sub­family B (P-glycoprotein), member 1 |
| ABCB2 | NM_000593 | -3.5±0.3 | Transporter 1 (TAP1), ATP ­binding cassette, sub­family B |
| ABCB3 | NM_000544 | -3.2±0.3 | Transporter 2 (TAP2), ATP ­binding cassette, sub­family B |
| ABCB4 | NM_000443 | 9.8±0.1 | ATP­ binding cassette, sub­family B (MDR), member 4 |
| ABCB6 | NM_005689 | 5.6±0.2 | ATP­ binding cassette, sub­family B (MDR), mitochondria ABC transporter (MTABC)3 |
| ABCC2 | NM_000392 | -8.6±0.1 | ATP­ binding cassette, sub­family C (MRP), member 2 |
| ABCC3 | NM_003786 | -7.2±0.1 | ATP­ binding cassette, sub­family C (MRP), member 3 |
| ABCC4 | NM_005845 | -10.4±0.2 | ATP­ binding cassette, sub­family C (MRP), member 4 |
| ABCD1 | NM_000033 | -3.4±0.3 | ATP­ binding cassette, sub­family D (ALD), member 1 |
| ABCD3 | NM_002858 | -13.0±0.1 | ATP­ binding cassette, sub­family D (ALD), member 3 |
| ABCD4 | NM_005050 | -4.8±0.1 | ATP­ binding cassette, sub­family D (ALD), member 4 |
| ABCG2 | NM_004827 | -12.0±0.1 | ATP­ binding cassette, sub­family G, member 2, breast cancer resistance protein (BRCP) |
| ABCG8 | NM_022437 | -13.8±0.1 | ATP­ binding cassette, sub­family G (WHITE), member 8, sterolin 2 |
| **SLC transporters** | | | |
| SLC2A1 | NM_006516 | -6.9±0.2 | Solute carrier family 2 (facilitated glucose transporter), member 1 |
| SLC2A2 | NM_000340 | -14.9±0.1 | Solute carrier family 2 (facilitated glucose transporter), member 2 |
| SLC2A3 | NM_006931 | -11.2±0.1 | Solute carrier family 2 (facilitated glucose transporter), member 3 |
| SLC3A1 | NM_000341 | -6.4±0.2 | Solute carrier family 3 (cystine, dibasic and neutral amino acid transporters, member 1 |
| SLC3A2 | NM_002394 | -11.7±0.1 | Solute carrier family 3 (activators of dibasic and neutral amino acid transport), member 2 |
| SLC7A5 | NM_003486 | -5.4±0.2 | Solute carrier family 7 (amino acid transporter light chain, L system), member 5 |
| SLC7A6 | NM_003983 | -3.4±0.3 | Solute carrier family 7 (amino acid transporter light chain, y+L system), member 6 |
| SLC7A7 | NM_003982 | -8.6±0.1 | Solute carrier family 7 (amino acid transporter light chain, y+L system), member 7 |
| SLC7A9 | NM_014270 | -16.1±0.1 | Solute carrier family 7 (glycoprotein­ associated amino acid transporter light chain, bo,+ system), member 9 |
| SLC7A11 | NM_014331 | -10.6±0.1 | Solute carrier family 7 (anionic amino acid transporter light chain, xc­ system), member 11 |
| SLC19A2 | NM_006996 | -11.6±0.1 | Solute carrier family 19 (thiamine transporter), member 2 |
| SLC22A3 | NM_021977 | -9.3±0.1 | Solute carrier family 22, organic cationic transporter (OCT) 3 |
| SLC22A7 | NM_006672 | -3.2±0.1 | Solute carrier family 22, organic anion transporter (OAT) 2 |
| SLC22A9 | NM_080866 | -15.1±0.3 | Solute carrier family 22, organic anion transporter (OAT) 7 |
| SLC28A1 | NM_004213 | -4.3±0.2 | Solute carrier family 28 (Na­-coupled nucleoside transporter), member 1 |
| SLC29A1 | NM_004955 | -6.3±0.2 | Solute carrier family 29 (nucleoside transporters), member 1 |
| SLC31A1 | NM_001859 | -18.0±0.1 | Solute carrier family 31, copper transporters (CTR) 1 |
| SLC38A2 | NM_018976 | -11.5±0.1 | Solute carrier family 38, member 2 |
| SLC38A5 | NM_033518 | -8.4±0.1 | Solute carrier family 38, member 5 |
| SLCO2B1 | NM_007256 | -4.8±0.2 | Solute carrier organic anion transporter family, member 2B1 |
| SLCO4A1 | NM_016354 | -3.2±0.3 | Solute carrier organic anion transporter family, member 4A1 |

ABC Transporters, ATP-Binding Cassette Transporters; SLC transporters; the Solute Carrier Transporters

**References**

# Choi K, Riviere JE, Monteiro-Riviere NA. Protein Corona Modulation of Hepatocyte Uptake and Molecular Mechanisms of Gold Nanoparticle Toxicity. Nanotoxicology. 2017;11:64-75.

# Monteiro-Riviere NA, Inman AO, Zhang LW. Limitations and relative utility of screening assays to assess engineered nanoparticle toxicity in a human cell line. Toxicol Appl Pharmacol. 2009;234:222-35.

# Chandran, P, Riviere JE, Monteiro-Riviere NA. Surface chemistry of gold nanoparticles determines the biocorona composition impacting on cellular uptake, toxicity and gene expression in human endothelial cells. Nanotoxicology. 2017;11:507-19.

# Cho EC, Xie J, Wurm PA, Xia Y. Understanding the role of surface charges in cellular adsorption versus internalization by selectively removing gold nanoparticles on the cell surface with a I_2_/KI etchant. Nano Lett. 2009;9:1080-4.

# Allabashi R, Stach W, de La Escosura-Muňiz A, Liste-Calleja L, Merkoci A. ICP-MS: a powerful technique for quantitative determination of gold nanoparticles without previous dissolving. J Nanopart Res. 2009;doi:10.1007/s11051-008-9561-2.

# Choi K, Ortega MT, Jeffery B, Riviere JE and Monteiro-Riviere NA. Oxidative Stress Response in Canine In Vitro Liver, Kidney and Intestinal Models with Seven Potential Dietary Ingredients. Toxicol. Lett. 2016;241:49-59.
